# Mesenchymal stem cell-derived extracellular vesicles exert pro-angiogenic and pro-lymphangiogenic effects in ischemic tissues by transferring various microRNAs and proteins including ITGa5 and NRP1

**DOI:** 10.1186/s12951-024-02304-y

**Published:** 2024-02-12

**Authors:** Anna Łabędź-Masłowska, Luisa Vergori, Sylwia Kędracka-Krok, Elżbieta Karnas, Sylwia Bobis-Wozowicz, Małgorzata Sekuła-Stryjewska, Michał Sarna, Ramaroson Andriantsitohaina, Ewa K. Zuba-Surma

**Affiliations:** 1 Department of Cell Biology, Faculty of Biochemistry, Biophysics and Biotechnology, Jagiellonian University, Krakow, Poland; 2 INSERM U1063, Oxidative Stress and Metabolic Pathologies, Angers University, Angers, France; 3 Department of Physical Biochemistry, Faculty of Biochemistry, Biophysics and Biotechnology, Jagiellonian University, Krakow, Poland; 4 Laboratory of Stem Cell Biotechnology, Malopolska Centre of Biotechnology, Jagiellonian University, Krakow, Poland; 5 Department of Biophysics, Faculty of Biochemistry, Biophysics and Biotechnology, Jagiellonian University, Krakow, Poland; 6 PhyMedExp, INSERM U1046, UMR CNRS 9214, Université de Montpellier, Montpellier, France

**Keywords:** Extracellular vesicles, Mesenchymal stem cells, Ischemic tissue regeneration, Angiogenesis, Lymphangiogenesis

## Abstract

Mesenchymal stem cells/stromal cells (MSCs)-derived extracellular vesicles (EVs) mediate pro-regenerative effects in damaged ischemic tissues by regulating angiogenesis. MSCs-EVs modulate functions of cells including endogenous mature cells, progenitors and stem cells, resulting in restoration of blood flow. However, the mechanisms underlying such MSC-EV activity still remain poorly understood. The present study analyzes biological effects of bone marrow (BM) MSC-EVs on endothelial cells (ECs) in ischemic tissues both in in vitro and in vivo conditions and elucidates the molecular mechanisms underlying the tissue repair. MSC-EVs were isolated from murine BM-derived MSCs and their morphological, antigenic and molecular composition regarding protein and microRNA levels were evaluated to examine their properties. Global proteomic analysis demonstrated the presence in MSC-EVs of proteins regulating pro-regenerative pathways, including integrin α5 (Itgα5) and neuropilin-1 (NRP1) involved in lymphangiogenesis. MSC-EVs were also enriched in microRNAs regulating angiogenesis, TGF-β signaling and processes guiding cellular adhesion and interactions with extracellular matrix. The functional effects of MSC-EVs on capillary ECs in vitro included the increase of capillary-like tube formation and cytoprotection under normal and inflammatory conditions by inhibiting apoptosis. Notably, MSC-EVs enhanced also capillary-like tube formation of lymphatic ECs, which may be regulated by Itgα5 and NRP1. Moreover, in a mouse model of critical hind limb ischemia, MSC-EVs increased the recovery of blood flow in ischemic muscle tissue, which was accompanied with increased vascular density in vivo. This pro-angiogenic effect was associated with an increase in nitric oxide (NO) production via endothelial NO-synthase activation in ischemic muscles. Interestingly, MSC-EVs enhanced lymphangiogenesis, which has never been reported before. The study provides evidence on pro-angiogenic and novel pro-lymphangiogenic role of MSC-EVs on ECs in ischemic tissue mediated by their protein and miRNA molecular cargos. The results highlight Itgα5 and NRP1 carried by MSC-EVs as potential therapeutic targets to boost lymphangiogenesis.

**Graphical Abstract:**

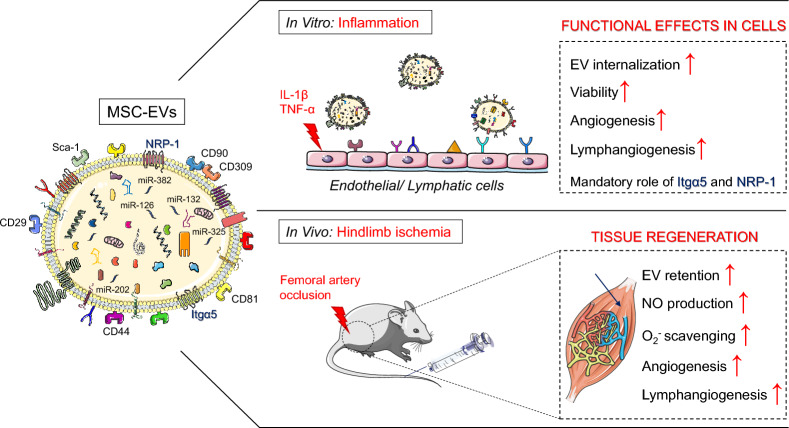

**Supplementary Information:**

The online version contains supplementary material available at 10.1186/s12951-024-02304-y.

## Introduction

Ischemic tissue injuries and diseases accompanied with progressing adverse impact on the patients functional condition, became a leading and growing cause of disabilities and deaths worldwide [1]. One of the key aspects in the treatment of such diseases include restoration of blood flow in injured tissues via inducing angiogenesis [2]. Since pharmacological and surgical treatments are insufficient to stimulate vascular remodeling, it has been believed that stem cells (SCs)-based therapies may provide a novel approach for ischemic tissue repair [2]. Mesenchymal stem/stromal cells (MSCs) exhibiting capacity to self-renewal and multi-lineage differentiation potential, are emerging as promising and safe therapeutic cells for ischemic diseases, which was confirmed in several studies [3]. Nowadays, close to 1500 clinical trials has been registered in the *ClinicalTrials.gov* database as studies using MSCs of various origin in the treatment of several human tissue injuries. The pro-regenerative effects observed following the MSC transplantation may be a result of their: (i) differentiation into mature cells of certain tissue, including into endothelial cells, (ii) immunomodulatory properties, (iii) paracrine activity including the release of pro-angiogenic and cytoprotective agent and (iv) extracellular vesicles (EVs) production [4, 5]. Since the retention of transplanted MSCs in injured tissues is relatively low [6, 7], the paracrine activity and EV release may predominantly drive the structural and functional recovery of injured tissues observed following their administration. These has recently increased interest in cell-free therapies employing EVs, which may carry bioactive compounds produced by their parental cells including SCs such as MSCs [8]. EVs play an important role in cell-to-cell communication ensuring an exchange of bioactive compounds between different cells and affecting their functions [9]. Growing number of reports indicate that EVs secreted by various types of SCs may modulate functions of other cells including not only endogenous mature cells, but also progenitors and stem cells residing in tissues, thereby improving regenerative processes in injured organs [9, 10]. Importantly, EVs are smaller than cells, which minimize a risk of blocking of small capillaries and vascular embolism after *i.v.* injection. It has been shown that bone marrow MSC-derived EVs may promote tissue repair by stimulating angiogenesis in vitro and in vivo [11, 12], however the mechanisms of their action are still not fully explored. Importantly, it remains an open question if MSC-EVs may stimulate other pro-regenerative mechanisms in ischemic tissues such as lymphangiogenesis representing other arm of tissue vasculature development.

Although single studies showing the molecular characteristics of human MSC-EVs at the protein [13] or microRNA level [14] have been reported, there are no results presenting an in-depth multi-faceted analysis of MSC-EVs strictly correlated with EVs potential biological activity relevant for ischemic tissue repair. Thus, in this study, we meticulously analyzed the molecular composition of murine MSC-EVs in the context of their functional interaction with target cells critical for ischemic tissue recovery such as endothelial cells in vitro. Importantly, we for the first time demonstrate here a crucial role of VEGF-C–Itgα5 and VEGF-C–NRP1 axes in promoting processes of lympangiogenesis in vitro. Moreover, we evaluated the pro-regenerative potential of systematically/intravenously (*i.v*) administrated MSC-EVs in murine model of ischemic tissue injury such as hindlimb ischemia (HLI) in vivo*.* We indicated the potential mechanisms of action of MSC-EVs based on their molecular composition and effects observed in tissues at histological and biochemical level after their administration in vivo. Besides angiogenic potential of MSC-EVs, we examined also an impact of MSC-EVs on lymphangiogenesis in vivo, which has never been described during regeneration following MSC-EVs treatment. Since there is a growing interest in developing novel EV-based approaches in the treatment of various tissue injuries (following e.g. stroke, myocardial infarction, burns), the results presented in this study may greatly contribute to this field indicating the future direction for the development of novel pro-angiogenic cell-free medicinal products.

## Materials and methods

The extended materials and methods are available in Additional file 1.

### Animals

Two strains of 4 to 8 week old mice were used in this study: (1) C57Bl/6 wild-type (WT) strain and (2) transgenic eGFP-expressing mice (C57Bl/6-Tg(GFP)J). All procedures, including the isolation of mouse tissues, were performed at the Jagiellonian University (JU) in Krakow in accordance with the approval of the Ethical Committee for Animal Experiments at the JU (approval number: 183/2012). Tissue regeneration and in vivo biodistribution experiments were performed at the Université d'Angers in Angers, France, in accordance with the guidelines and authorization of the French Ministry of Agriculture, based on the European Community standards for the care and use of laboratory animals.

### Cell culture

#### Isolation and culture of murine BM-derived MSCs

Murine bone marrow (BM) cells were harvested from tibiae and femurs by rinsing the bone cavities with Dulbecco’s modified Eagle’s medium/nutrient mixture F-12 ham (DMEM/F12; #D8437, Merck). BM-derived cells were centrifuged (350×*g*, 6 min, RT), resuspended in complete culture medium-DMEM/F12 supplemented with 10% fetal bovine serum (FBS; #F2442, Merck), 100 U/mL penicillin, and 100 µg/mL streptomycin (P/S; Gibco, ThermoFisher Scientific)-and seeded into tissue culture flasks (BD Falcon, Corning). The rinsed bones were fragmented and enzymatically digested with a mixture of collagenase type I and II (each at a concentration of 1 mg/mL; Merck) for 1.5 h at 37 °C. Collagenases were inactivated by adding DMEM/F12 supplemented with 10% FBS, and cells were then centrifuged (350×*g*, 6 min, RT), resuspended in complete culture medium, and transferred to culture flasks containing previously rinsed BM-derived cells. Cells were cultured for 72 h and non-adherent cells were removed. Passaging was performed with 0.25% trypsin/EDTA (Gibco, ThermoFisher Scientific) when cell confluence reached approx. 90%. Cells with the highest viability (> 98%) were used for further experiments including EV harvest.

#### Culture of MCECs

Mouse cardiac endothelial cells (MCECs) were purchased from CELLutions Biosystems Inc. (#CLU510, Ontario, Canada) and cultured in Dulbecco’s modified Eagle’s medium (DMEM; #D5796, Merck) supplemented with 10 mmol/L HEPES (HyClone, GE Healthcare Life Sciences) and 5% FBS (#F2442, Merck) on 0.1% gelatin-coated cell culture dishes (BD Falcon, Corning). Cells were passaged with Tryple Select Enzyme 1× (ThermoFisher Scientific) after reaching a confluence of app. 80–90%.

#### Culture of LECs

Mouse primary dermal lymphatic endothelial cells (LECs) from the C57BL/6 mouse strain were purchased from Cell Biologics (#C57-6064L, Chicago, USA) and cultured in tissue culture flasks precoated with gelatin-based coating solution (#6950, Cell Biologics) in Culture Complete Mouse Endothelial Cell Medium (#M1168, Cell Biologics). Cells were passaged after reaching a confluence of approx. 80%.

### Isolation of EVs

Murine MSC-derived extracellular vesicles (MSC-EVs) were isolated from cell culture supernatants of BM-derived MSCs (at passages 4, 5, or 6) using a sequential centrifugation method including a double ultracentrifugation step. Briefly, MSCs reaching 80% confluence were washed with PBS (w/o Ca2+, Mg2+, HyClone) and cultured in ultracentrifuged medium DMEM/F12 containing 10% FBS (to remove EVs and other small particles of FBS origin) for 48 h. The collected cell-conditioned media were centrifuged at 350×*g* for 10 min at 4 °C to remove individual detached cells. The supernatants were transferred to new tubes (Sarstedt) and centrifuged at 500×*g* for 10 min at 4 °C to remove residual cells and cell debris. The supernatants were then immediately processed or frozen at − 80 °C. In the next step, the fresh or frozen/thawed cell-conditioned media were centrifuged at 2000×*g* for 20 min at 4 °C to remove small cellular debris and apoptotic bodies. Finally, EVs (and large protein/RNA complexes) were collected by ultracentrifugation of the supernatants in PP Oak Ridge tubes (Thermo Fisher) at 100,000×*g* for 75 min at 4 °C (in a T-865 fixed angle rotor, Thermo Fisher; in an Optima XPB ultracentrifuge, Beckman Coulter), washed once in ultrapure PBS (#BE17-512F, Lonza) and pelleted (centrifuged at 100,000×*g* for 75 min at 4 °C using the rotor and centrifuge described above). Freshly isolated MSC-EVs were resuspended in PBS (#BE17-512F, Lonza) and transferred to Protein LoBind Tubes (Eppendorf). Protein content of MSC-EV samples was quantified by the Bradford assay.

### Particle size analysis

Tunable resistive pulse sensing (TRPS, qNano, Izon) was used to analyze the size distribution of nanoparticles in MSC-EV samples using NP200 nanopore (analytical range 85–500 nm; Izon Science) at 20 or 10 mbar pressure. Samples were analyzed for 5 min or until 1000 particles were counted. Data processing and analysis were performed using Izon Control Suite software v2.2 (Izon Science).

### Analysis of EV morphology

To analyze the morphology of MSC-EVs according to the recommendations published by the International Society for Extracellular Vesicles (ISEV) [15], Atomic Force Microscopy (AFM) technique was used. Images of the topography of the mika surface containing attached MSC-EVs were captured using the PeakForce Tapping (PFT) mode.

### Flow cytometric analysis of EV antigenic profile

Surface markers on MSC-EVs were analyzed using Apogee A50-Micro flow cytometer (Apogee Flow Systems, UK). The following fluorochrome-conjugated antibodies against murine antigens were used according to the manufacturer’s protocols: CD29-APC (clone: HMβ1-1, Biolegend), CD44-APC (clone: IM7, Biolegend), CD81-APC (clone: Eat2, BD Bioscence), CD90-APC (clone: 30-H12, Biolegend), CD309-APC (clone: Avas12, Biolegend) and Sca-1-APC (clone: E13-161.7, Biolegend) as well as the following isotype controls: Armenian hamster IgG-APC (clone: HTK888, Biolegend), Rat IgG2a, κ-APC (clone: RTK2758, Biolegend) and rat IgG2b, κ-APC (clone: RTK4530, Biolegend). MSC-EVs were co-stained with SYTO RNA Select dye (ThermoFisher Scientific), which binds RNA molecules. Staining was conducted for 30 min in the dark at 4 °C. The obtained results were analyzed using Apogee Histogram software (Apogee Flow Systems). In order to confirm the presence of the indicated antigens on MSC-EVs, an ImageStream X Mark II imaging cytometer (Merck) was additionally used.

### Western blotting

Western blotting was used to analyze the presence of EV-specific markers in the isolated MSC-EV samples. Briefly, proteins were separated by SDS-PAGE and transferred to PVDF membranes (Merck). The blocked PVDF membranes were immunolabeled with the following antibodies: rabbit anti-mouse CD9 (1:1000, AntibodyGenie), goat anti-mouse calnexin (1:500, ThermoFisher Scientific), rabbit anti-mouse syntenin (1:1000, ThermoFisher Scientific), or mouse anti-mouse β-actin (1:2000, ThermoFisher Scientific) overnight at 4 °C. Horseradish peroxidase-conjugated goat anti-rabbit (1:4000, Dako), rabbit anti-goat (1:4000, ThermoFisher Scientific), or goat anti-mouse (1:4000, ThermoFisher Scientific) antibodies were then incubated with PVDF membranes for 1 h. Product detection was performed using Supersignal West Dura chemiluminescent substrate (Pierce). Imaging of specific bands was performed using a ChemiDoc MP system (Bio-Rad) and Image Lab software (version 5.1, Bio-Rad).

### Global proteomic analysis of EV content

Lysed MSC-EV samples were prepared for global proteomic analysis by liquid chromatography-tandem mass spectrometry (LC–MS/MS) using the filter-assisted sample preparation (FASP) method described by Wisniewski et al. [16]. The obtained peptides were then fractionated according to the protocol also described by Wisniewski et al*. *[17]. The MSC-EV-derived peptides were analyzed by mass spectrometry (MS) using high-resolution Q-Exactive mass spectrometer (Thermo Scientific) coupled to an UltiMate 3000RS LC nanoSystem (Thermo Scientific). In the experiment performed, 3 samples (biological replicates) of MSC-EVs were profiled. The data obtained from LC–MS/MS data were analyzed using Proteome Discoverer 1.4 (Thermo Scientific). Briefly, an in-house MASCOT server (v. 2.5.1, Matrix Science, Boston, MA, USA) was used for searching against the Swissprot_201509 database, which was restricted to the *Mus musculus* taxonomy. Validation of the search results was performed in Proteome Discoverer 1.4 using the Percolator algorithm according to Kall et al*.* [18]. The false discovery rate (FDR) was set below 1%.

The total number of proteins identified was more than 2200. Only proteins identified in at least 2 of the 3 MSC-EV samples, based on 2 or more unique peptides for each protein, were considered for further analysis. For the proteins identified in MSC-EVs, GO enrichment analysis was performed in DAVID Functional Annotation Bioinformatics Microarray Analysis (https://david.ncifcrf.gov/home.jsp). A p-value threshold of 0.05 was used in the analysis. In addition, proteins identified in MSC-EVs were also analyzed for their association with specific pathways based on GO: Panther Pathways annotation, using FDR below 5% or 1%. In the next step, interaction analysis was performed on the proteins identified in MSC-EVs using the commercially available STRING Ver. 10.5 application (https://string-db.org/). In addition, the ExoCarta database [19] was used to confirm or exclude the presence of the top 100 proteins frequently identified in small EVs (previously called exosomes).

### Analysis of microRNA content in EVs

Real-time qRT-PCR profiling was used to determine the microRNA content of MSC-EVs. Briefly, total RNA (including small RNAs) was purified from MSC-EV samples using miRCURY RNA Isolation Kit—Cell & Plant (Exiqon) according to the manufacturer’s protocol. Samples were treated with Turbo DNAse (according to the manufacturer’s instructions; Ambion, ThermoFisher Scientific) to ensure complete removal of genomic DNA contamination. The purity and RNA concentration were determined using a Nano Photometer (Implen). Only RNA preparations with a concentration greater than 5 ng/μL and a purity of approximately 2.00, as measured by the absorbance ratio A260/280, were used for further analysis. Next, cDNA synthesis was performed using the Universal cDNA Synthesis Kit II (Exiqon) according to the manufacturer’s instructions in a C1000 Touch Thermal Cycler (Bio-Rad). The cDNA products were then diluted and transferred to the Ready-to-use Mouse and Rat microRNA (miRNA) PCR Panel I + II, V3.M (Exiqon) and quantified using real-time qPCR based on Power SYBR Green Master Mix (Applied Biosystems, ThermoFisher Scientific) and locked nucleic acid (LNA)-enhanced miRNA-specific primers. The qPCRs were run on the QS6 Real-Time PCR System (ThermoFisher Scientific) using the reaction parameters recommended by Exiqon. The data were analyzed using GeneEx software (bioMCC, Freising, Germany) and further using mirPath v.3 (DIANA-Lab, University of Thessaly, Greece) dedicated to the assessment of miRNA regulatory roles and the identification of controlled pathways as described by Vlachos et al. [20]. Three (3) independent samples (biological replicates) of MSC-EVs were analyzed in this study. For the most enriched miRNAs in MSC-EVs (fold change in expression ≥ 2.0 when compared to parental MSCs), the heat-map showing their involvement in functional pathways was generated with DIANA mirPath web tool.

### Internalization of EVs by endothelial cells in vitro

#### Fluorescent labeling of MSC-EVs

To examine MSC-EV uptake by target cells such as vascular endothelial cells in vitro*,* MSC-EV samples were stained with lipophilic Vibrant DiO Cell-Labeling Solution (ThermoFisher Scientific) and SYTO RNA Select green fluorescent cell stain (ThermoFisher Scientific) according to the manufacturer’s protocols to label membrane lipids and RNA within the vesicles, respectively. MSC-EVs were stained for 20 min in the dark at RT and freshly used for experiments.

#### Analysis of cellular uptake of MSC-EVs

MCEC cells were seeded on glass bottom dishes (Ø 35 mm, WillCo Wells B.V.) at a density of 5 × 10^4^ cells/dish in complete DMEM medium (#D5796, Merck) supplemented with 5% FBS (#F2442, Merck). After 24 h post seeding, cells were washed with PBS (w/o Ca^2+^, Mg^2+^, HyClone) and divided into two groups:

1. Control—further culture of MCECs in complete DMEM medium with 5% FBS for the following 24 h;
2. Microenvironment mimicking inflammation and hypoxia—further culture of MCECs in complete DMEM medium with 1% FBS, 10 ng/mL IL-1β (#211-11B, Peprotech) and 10 ng/mL TNF-α (#315-01, Peprotech) for the following 24 h.

Fluorescently-labelled MSC-EVs (50 µg) were then added into each dish and MCECs were further incubated at 37 °C in humidified atmosphere containing 5% CO_2_ and 21% O_2_ (Control) or 5% CO_2_ and 2% O_2_ (microenvironment of inflammation and hypoxia). Internalization of MSC-EVs by MCEC cells was examined after 2, 4 and 24 h of incubation with EVs by using Leica DMI6000B inverted fluorescence microscope (ver. AF7000, Leica Microsystems) equipped with Leica Application Suite X software (Leica Microsystems). The mean fluorescence intensity of MCECs was measured using ImageJ software (Maryland, USA).

### Capillary-like tube formation assay in vitro

#### Assessment of pro-angiogenic potential of MCECs

To investigate an effect of MSC-EVs on the pro-angiogenic potential of vascular endothelial cells—MCECs, capillary formation was assessed in vitro*.* Briefly, 24-well plates (BD Falcon, Corning) were coated with Matrigel Matrix Grow Factor Reduced (100 µL/well, Corning) and incubated at 37 °C for 30 min. MCECs were seeded at a density of 5 × 10^4^ cells/well in the EBM-2 basal medium supplemented with EGM-2MV SingleQuots Kit (both from Clonetics, Lonza). The seeded MCECs were divided into the following four subgroups:

1. Control—further incubation of MCECs in EBM-2 supplemented with EGM-2MV SingleQuots Kit;
2. Microenvironment of inflammation and hypoxia—further incubation of MCECs in EBM-2 supplemented with EGM-2MV SingleQuots Kit, 10 ng/mL IL-1β (#211-11B, Peprotech) and 10 ng/mL TNF-α (#315-01, Peprotech);
3. Control + MSC-EVs—further incubation of MCECs in EBM-2 supplemented with EGM-2MV SingleQuots Kit and 10 µg/mL MSC-EVs;
4. Microenvironment of inflammation and hypoxia + MSC-EVs—further incubation of MCECs in EBM-2 supplemented with EGM-2MV SingleQuots Kit, 10 ng/mL IL-1β (#211-11B, Peprotech), 10 ng/mL TNF-α (#315-01, Peprotech) and 10 µg/mL MSC-EVs.

Capillary-like structure formation by MCECs was examined and recorded at 37 °C in a 5% CO_2_ chamber (PeCon GmbH, Erbach, Germany) mounted on a Leica DMI6000B inverted microscope equipped with a dry 10×, NA 0.25 objective, integrated modulation contrast optics and a DFC360FX digital CCD camera (Leica Microsystems). The capillary-like tube formation of MCECs was time-lapse recorded for 8 h using Leica Application Suite X software (Leica Microsystems). The total tube length was quantified using the Image J software.

#### Assessment of pro-lymphangiogenic potential of LECs

The formation of three-dimensional lymphatic capillary-like structures by endothelial cells of lymphatic vessels—LECs treated with MSC-EVs was examined by performing a Matrigel-based tube formation assay. Each well of a µ-slide (ibidi GmbH) was filled with 10 µL of ECMgel (#E1270, Sigma ECMgel, Merck), which was allowed to polymerize for 30 min at 37 °C. The LECs were seeded at a density of 1 × 10^4^ cells/well and cultured in endothelial medium in the presence or absence of 100 ng/mL VEGF C (#SRP4634, Merck). The LECs were treated with MSC-EVs and/or recombinant murine IL-1β (10 ng/mL) (#211-11B, Peprotech), recombinant murine TNF-α (10 ng/mL) (#315-01, Peprotech) to induce inflammatory conditions. In another set of experiments, mouse/rat anti-neuropilin-1 antibody (#AF566, R&D System) and anti-integrin alpha 5 (CD49e) antibody (#103908, BioLegend) were preincubated with MSC-EVs for 1 h at 4 °C, and then centrifuged to remove excess of blocking antibody, before being added to the cells. A positive control was performed with 20 ng/mL PMA (#P8139, Merck). After 18 h of incubation, the tube formation images were captured at fourfold magnification using the Olympus CK40 (Olympus CK40, Olympus Corporation). The total tube length and number of tubes were quantified using the Image J software.

### Expression of EC activation markers in vitro

To investigate the level of expression of markers of activated vascular endothelial cells under different conditions, immunofluorescence staining and flow cytometry analysis of cells were performed.

Briefly, MCECs were seeded at a density of 10^5^ cells/dish (Ø 60 mm, Falcon, Corning) in complete DMEM medium (#D5796, Merck) supplemented with 5% FBS (#F2442, Merck). Cells were washed with PBS (w/o Ca^2+^, Mg^2+^, HyClone) after 24 h and divided into two groups:

1. Control—further culture of MCECs in complete DMEM with 5% FBS for the following 24 h;
2. Microenvironment mimicking inflammation and hypoxia—further culture of MCECs in DMEM with 1% FBS, 10 ng/mL IL-1β (#211-11B, Peprotech) and 10 ng/mL TNF-α (#315-01, Peprotech) for the following 24 h.

The following factors were then added to MCECs incubated in the inflammatory and hypoxic microenvironment: 10 ng/mL IL-1β, 10 ng/mL TNF-α or 10 ng/mL IL-1β, 10 ng/mL TNF-α and 10 µg/mL MSC-EVs. Cell culture medium was removed after 4 or 24 h of incubation and MCECs were washed with PBS (w/o Ca^2+^, Mg^2+^, HyClone), and passaged. Cells were further centrifuged (300×*g*, 5 min, RT), resuspended in staining medium (DMEM supplemented with 1% FBS) and stained with the following fluorochrome-conjugated anti-mouse antibodies: CD54-APC (clone: YN1/1.7.4, Biolegend), CD62E-PE (clone: 10E9.6, BD Bioscence), CD62P-Alx647 (clone: RB40.34, BD Bioscence), CD106-APC (clone: 429, Biolegend) for 30 min in the dark at 4 °C. Samples were then washed with PBS (w/o Ca^2+^, Mg^2+^, HyClone), centrifuged (300×*g*, 6 min, RT) and resuspended in staining medium. DAPI (3 μM, ThermoFisher Scientific) was added 10 min prior to analysis to exclude dead cells from further analysis. Cells were analyzed using LSR Fortessa flow cytometer (Becton Dickinson).

### Murine model of hind limb ischemia (HLI) in vivo

To induce unilateral hind limb ischemia (HLI), the ligation of the left femoral artery (FA) was performed according to the procedure described by Niiyama et al*.* established at the Stanford University, CA, USA [21]. In the male C57BL/6 mice (Charles River Laboratories, L'Arbresle, France) at the aged of 6 to 8 weeks, the distal and proximal ends of the FA were occluded with 6–0 silk suture (Ethicon). The segment of FA between the distal and proximal knots were carefully transected. The wound was then closed and dressed. Mice were randomly divided into two subgroups (N = 8–9/group): (1) vehicle (PBS, control group) and (2) MSC-EVs (EV-treated group). Respectively, PBS or MSC-EVs (10 µg/mL of blood), respectively, were administered intravenously through the tail vein every 3 days for 21 days of the experiment.

The total blood volume for each mouse was calculated based on the consideration that the total blood volume in the mouse is in the range of 6–8 mL/100 g of body weight [22] as shown in the Additional file 1: Table S1. At 7, 14 and 21 days after FA occlusion, blood flow was measured in both limbs as described below. At 21 days after HLI, animals were sacrificed and muscle tissues were harvested for further biochemical and histological analysis. The in vivo experimental design is presented in Additional file 1: Fig. S1.

### Analysis of blood flow in tissues in vivo

To provide functional evidence of ischemia and further recovery of blood perfusion, laser Doppler perfusion imaging technique based on dynamic light scattering in tissue was used [23]. Briefly, mice were anesthetized with Aerrane Isoflurane (Baxter) and placed on a heating plate to maintain a stable cutaneous temperature in order to minimize temperature variation throughout the experiments. Limb perfusion was measured using a laser Doppler flow probe (PF 408, Perimed). A minimum of 3 flow measurements were performed per mouse. Limb blood flow was expressed as the ratio of left (ischemic) to right (non-ischemic) limb perfusion, as described by Limbourg et al. [24].

### Analysis of EV biodistribution in vivo

To investigate the retention of MSC-EVs in different organs after i.v. administration, animal tissues were analyzed using an in vivo fluorescence imaging system. Briefly, male C57BL/6 mice were injected with 100 μg GFP-positive MSC-EVs or PBS via the tail vein 24 h after HLI. Mice were sacrificed 4 or 24 h after injection of MSC-EVs or PBS. Blood, liver, spleen, lung, kidney, and limb were then isolated. Multispectral imaging system MAESTRO In-Vivo Fluorescence Imaging System (Cambridge Research & Instrumentation; Woburn, MA, USA) was used to analyze the biodistribution of GFP-positive MSC-EVs. For plasma isolation, blood was centrifuged at 350×*g*, 7 min, RT and transferred to a new 1.5 mL tube. The respective fluorescence of isolated organs, plasma and limbs was analyzed using the MAESTRO In-Vivo Fluorescence Imaging System software (Cambridge Research & Instrumentation; Woburn, MA USA).

### Immunofluorescence staining and confocal microscopy

To prepare frozen sections for semiquantitative analysis of neovascularization in MSC-EV-treated tissues, both ischemic and non-ischemic gastrocnemius muscles were dissected and embedded in Tissue-Tek O.C.T compound (Sakura Finetek), frozen in liquid N_2_ and stored at − 80 °C until further processing. Frozen tissues were sectioned using Leica CM3050S cryostat (Leica Biosystems). Subsequently, cryosections (7 µm) were fixed in 100% methanol (Merck) for 5 min at − 20 °C, and non-specific binding sites were saturated with blocking buffer containing 5% BSA (Euromedx) in PBS for 1.5 h at RT. Tissue sections were then incubated with rat anti-mouse CD31 antibody (marker of blood vessel endothelial cells of s; 1:100, BD Biosciences) and rabbit anti-mouse Lyve1 antibody (marker of lymphatic vessel endothelial cells; 1:200, Acris Antibodies) overnight at 4 °C. The sections were then washed tree times with PBS and incubated (for 1 h at RT) with goat anti-rat FITC-conjugated IgG (1:100, Southern Biotech) to identify blood vessels or goat anti-rabbit Alexa546 (1:500, Interchim) secondary antibody to detect Lyve1 expression. To visualize nuclei, tissue sections were stained with DAPI (300 nM, Santa Cruz Biotechology) for 3 min at RT. After final washes, the coverslips were mounted. LSM 700 confocal (Zeiss) was used for the optical sectioning of the tissue sections. Digital imaging was performed using the Laser Sharp software. Vessels were quantified using ImageJ software and counted in at least five randomly selected fields for each muscle section, and the mean value for each section was calculated (magnification 40×).

### Analysis of NO and O_2_^−^ determination production in tissues

Detection of nitric oxide (NO) and superoxide anion (O_2_^−^) production in mouse tissues was performed using electronic paramagnetic resonance (EPR) with Fe^2+^ diethyldithiocarbamate (DETC) as a spin trap. At 21 days after FA ligation, animals were euthanized, and aortas and both ischemic and nonischemic muscles were dissected and incubated in Krebs-HEPES buffer containing Fe(DETC)_2_ solution for analysis of NO production or deferoxamine, DETC and CMH solution for analysis of O_2_^−^ production for 45 min at 37 °C.

The tissues for NO and O_2_^−^ production analysis were then immediately frozen in liquid N_2_. Measurement of NO and O_2_^−^ production was performed using a bench-top x-band spectrometer Miniscope (Magnettech, MS200, Berlin, Germany). Recordings were made at 77 °K using a Dewar flask. The instrument setting was as follow: 10 mW of microwave power, 1 mT of amplitude modulation frequency, 60 s of sweep time and 5 scans.

### Analysis of angiogenesis-related proteins in tissues

In each experiment, the aorta and the skeletal muscle from both ischemic and nonischemic limbs were removed and frozen in liquid N_2_ to further evaluate the expression of angiogenesis-related proteins after MSC-EV treatment. Briefly, homogenized tissue samples were separated by SDS-PAGE and transferred onto PVDF membranes (Merck). The blocked PVDF membranes were incubated overnight at 4 °C with one of the following primary monoclonal antibodies: anti-endothelial NOS (eNOS; 1:2500, BD Biosciences), anti-caveolin-1 (1000, BD Biosciences), anti-phospho-eNOS (Ser 1177, 1:1000, Cell Signaling Technology), anti-Akt (1:1000, Cell Signaling Technology), anti-phospho-Akt (Ser 473, 1:2000, Cell Signaling Technology), and anti-VEGF (1:1000, R&D Systems). A rabbit polyclonal anti-actin antibody (1:5000, Merck) was used to visualize protein loading on the gel. The membranes were then washed at least three times in Tris buffer containing 0.05% Tween-20 and incubated for 1 h at RT with the appropriate horseradish peroxidase (HRP)-conjugated secondary antibody (Amersham, Piscataway, NJ). The protein-antibody complexes were detected by ECL-Plus Chemiluminescence kit (Santa Cruz Biotechnology, Santa Cruz, CA) according to manufacturer’s protocol using Image Reader Las-3000 (Fujifilm).

### Statistical analysis

Data are presented as mean ± SD or SEM as indicated. Statistical analyses were performed by Student’s *t*-test or Mann–Whitney U-test (nonparametric) using Prism software package 8.00 or 5.00 (GraphPAD Software, San Diego, CA). *P* < 0.05 was considered to be statistically significant.

## Results

### MSC-EVs carry proteins and miRNAs that regulate pro-regenerative processes in injured tissues

**Fig. 1:**
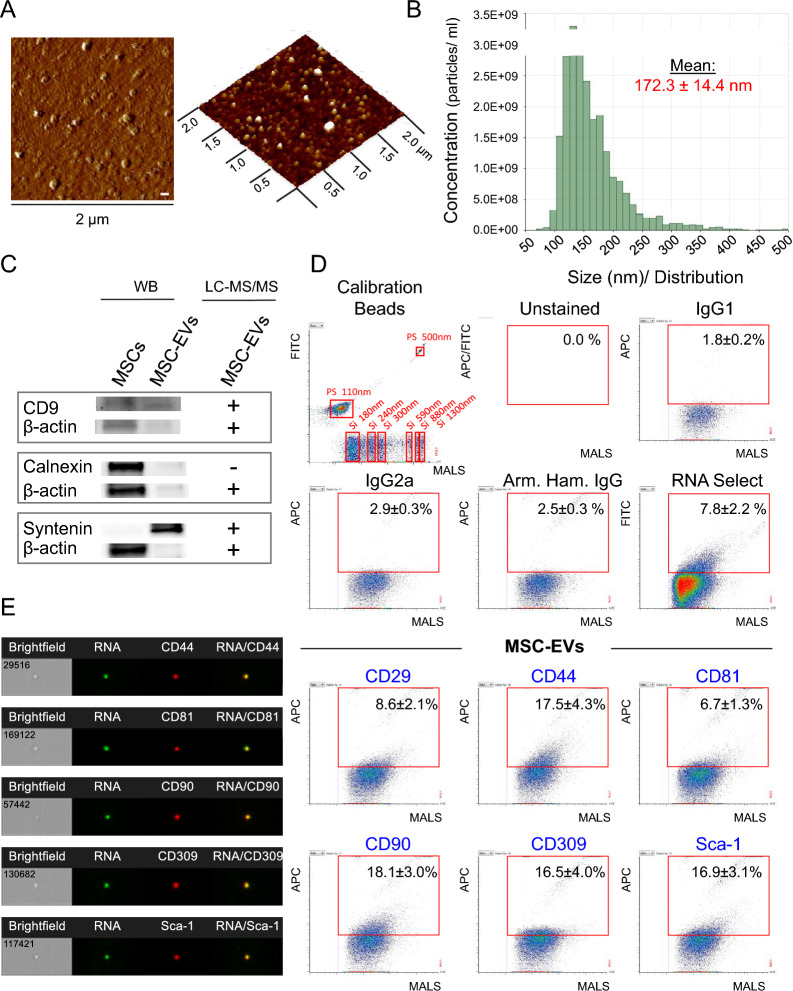
Characterization of MSC-EVs. **A** Representative images of MSC-EVs (left) and their 3D topography (right) by atomic force microscopy (AFM). Size of the scan 2 µm. Scale bar = 1.00 nm. **B** Representative histogram of particles size distribution in MSC-EVs specimen by IZON qNano. **C** Representative Western immunoblot confirming the presence of typical EVs markers (CD9 and syntenin) and lack of expression of endoplasmic reticulum protein-calnexin in MSC-EVs when compare to MSCs confirmed by mass spectrometry results (LC–MS/MS). **D** High-resolution flow cytometry analysis of MSC-EVs. (Top) Histogram showing the size distribution of mixed size synthetic beads (PS, fluorescently labeled polystyrene calibration beads detected in FITC channel; and Si, unlabeled silicone calibration beads) and representative histograms from analysis of control samples (unstained, isotype controls and labeled with RNA Select). (Bottom) Representative dot plots showing the medium-angle light scatter (MALS) related to EV size vs. fluorescence intensity indicating expression of selected EV-specific markers (CD81), MSC-specific antigens (CD29, CD44, CD90, Sca-1) antigens and VEGFR3 (CD309) on RNA-enriched MSC-EVs. **E** Analysis of selected markers on MSC-EVs by imaging cytometry

Following ISEV recommendations for EV characterization, we found that our isolates of murine bone marrow MSC-EVs represent heterogeneous populations of particles with size ranged between 70 and 430 nm and the mean diameter of 172.3 ± 14.4 nm (Fig. 1A, B). Analysis of basic protein content in our MSC-EV samples showed that they contained subpopulations of vesicles carrying parental cell specific antigens (e.g. CD29, CD44, CD90 and Sca-1) and tetraspanins (e.g. CD9, CD63 and CD81) (Fig. 1C–E, Additional file 1: Table S2) representing 1st category of proteins occurring in all EVs [15]. MSC-EVs contained also cytosolic proteins such as syntenin, annexins, actin, tubulin (Fig. 1C, Additional file 1: Table S2) representing 2nd category of proteins occurring in all EVs [15]. Interestingly, calnexin being major component of non-EV co-isolated structures [15] was not detected in EV specimens (Fig. 1C), whereas our MSC-EV population were enriched in small EVs (resembling mostly exosomes) as shown by the presence of 82 out of the top 100 most identified small EV marker proteins published in ExoCarta database (Additional file 1: Table S2) as well as 14 out of the 15 hallmark proteins identified by different investigators in MSC-EVs with smaller size (≤ 200 nm) (Additional file 1: Table S3). Subpopulation of MSC-EVs also carried VEGF receptor 2 (CD309) on their surfaces (Fig. 1D, E).

Extending the studies on molecular composition of MSC-EVs, we conducted further global proteomic analysis of MSC-EV content by mass spectrometry followed by bioinformatics analysis of the identified proteins. We identified more than 2200 proteins to be carried by MSC-EVs and only proteins identified in at least 2 out of the 3 MSC-EV samples based on 2 or more unique peptides were considered for further analyses.

**Fig. 2:**
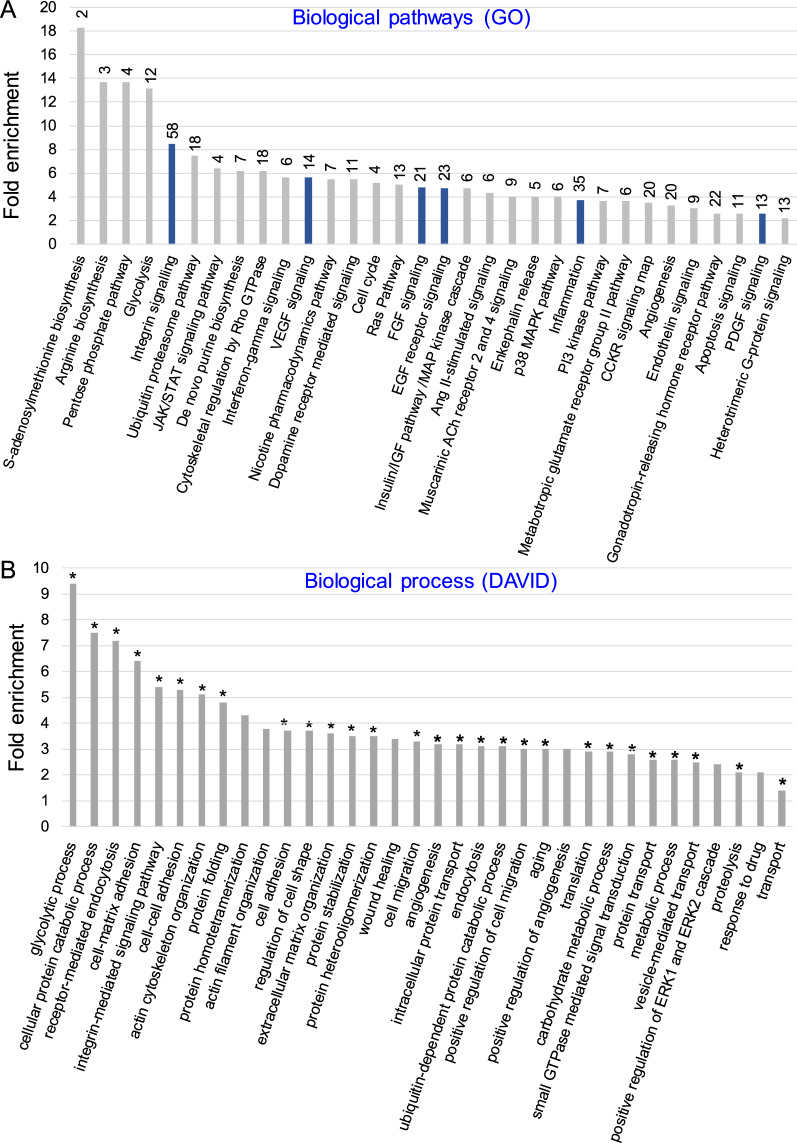
Global MSC-EV protein content and its role in biological processes and pathways. **A** Gene Ontology (GO) analysis. Panther pathway overrepresentation analysis of proteins identified in at least 2 out of 3 MSC-EV samples based on 2 or more unique peptides identified for every protein by LC–MS/MS. The results were displayed as fold enrichment of identified proteins in specific biological pathways. Total number of MSC-EV proteins identified for every pathway is presented over every bar. The navy bars represent regeneration-related pathways. Test type: Fisher’s Exact, Correction: FDR; P < 0.05, FDR < 0.05. **B** DAVID functional annotation bioinformatics microarray analysis. The analysis was conducted exclusively for proteins identified in at least 2 out of 3 MSC-EV samples based on 2 or more unique peptides. On the graph were displayed the most significant groups of proteins containing at least 20 proteins in terms. P < 0.05, Benjamini test < 0.05, Fisher Exact test < 0.05, (*) FDR < 0.05

With regard to *biological pathways*, the analysis conducted by Gene Ontology (GO) indicated the presence in MSC-EVs of multitude proteins involved in several signaling pathways including the ones known to play pro-regenerative role such as e.g. involved in regulation of inflammation, cell response after EGF, FGF, VEGF or PDGF binding, as well as angiogenesis (Fig. 2A). The analysis of a role of the identified proteins in *biological processes* (conducted by DAVID Functional Annotation Bioinformatics Microarray Analysis tool) revealed enrichment of MSC-EVs in proteins involved in or regulating e.g., metabolic processes such as glycolysis, protein catabolic processes, receptor-mediated endocytosis, cell-to-matrix and cell-to-cell adhesion, cell migration as well as pro-regenerative processes such as wound healing, angiogenesis and their positive regulation (Fig. 2B).

**Fig. 3:**
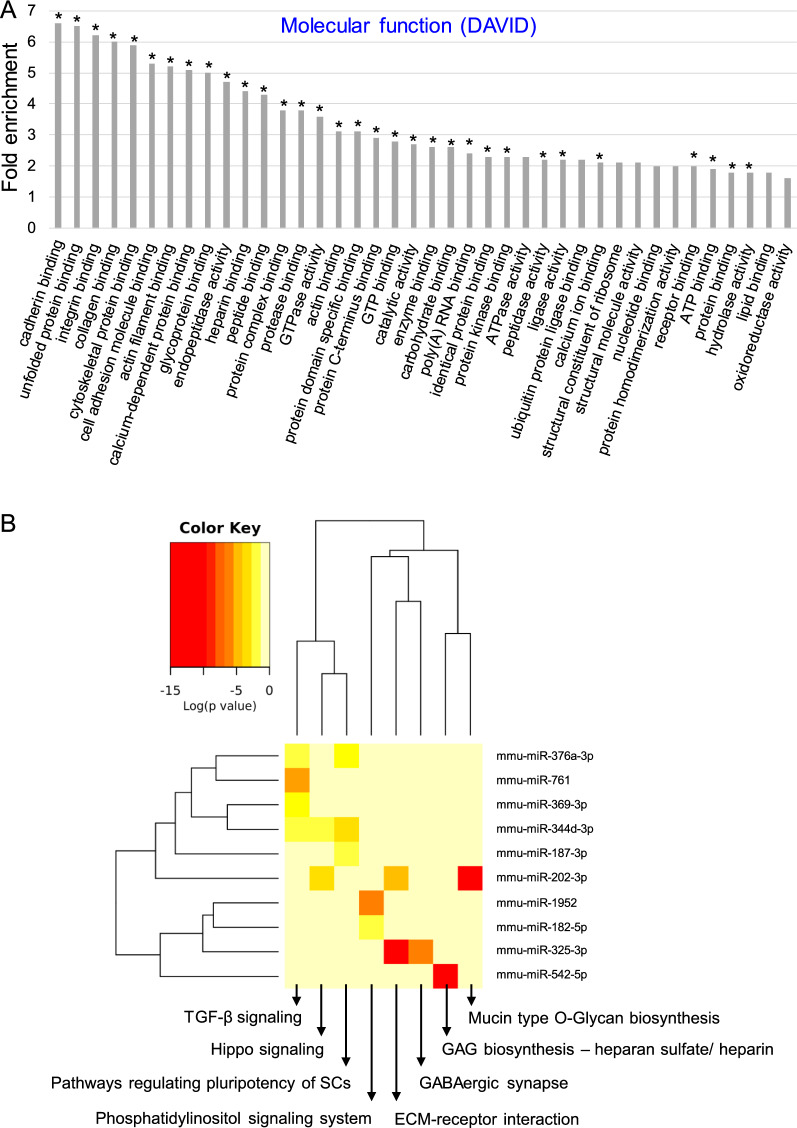
Molecular functions of proteins and microRNAs present in MSC-EVs. **A** Analysis of global proteomic content of MSC-EVs with focus on molecular functions by DAVID functional annotation bioinformatics microarray analysis. The analysis was conducted on proteins which were identified at least in 2 out of 3 MSC-EV samples based on 2 or more unique peptides. On the graph were displayed the most significant groups of proteins containing at least 20 proteins in terms. P < 0.05, Benjamini test < 0.05, Fisher Exact test < 0.05, (*) FDR < 0.05. **B** Heat-map generated with DIANA mirPath web tool showing functional pathways insertion for the most enriched miRNAs in MSC-EVs when compared to parental MSCs (fold change in expression ≥ 2.0). The attached dendrograms show hierarchical clustering results for miRNAs (Y-axis) and pathways (X-axis). Red squares indicate lower P values (more significant) and higher interaction of each miRNA with specific molecular pathway. P < 0.05

With reference to *molecular function*, MSC-EVs carried a wide range of proteins essential for interactions and binding several other proteins, ions, DNA, RNA, ECM or cell adhesion molecules (Fig. 3A). The proteins identified in MSC-EV samples and indicated by DAVID Functional Annotation Bioinformatics Microarray Analysis tool as involved in several cellular processes as well as those involved in cell adhesion have also been grouped in the STRING database (Search Tool for the Retrieval of Interacting Genes/Proteins). These analyses revealed a clear network of connections and interaction between these proteins (Additional file 1: Fig. S2) indicating their functional importance for MSC-EV activity. Moreover, extensive interaction network was also detected between proteins involved in cell adhesion, angiogenesis and wound healing (Additional file 1: Fig. S3). Among these proteins, we identified integrin α5 (Itgα5) involved in inflammatory lymphangiogenesis [25] and neuropilin-1 (NRP1) which acts as a key modulator of vascular, lymphatic, and inflammatory cell responses [26]. Taken together, the in-depth proteomic analysis revealed that MSC-EVs carry proteins that play a significant role in the regulation of multiple cellular processes including those associated with pro-regenerative events in injured tissues.

We further characterized protein contents of MSC-EVs with reference to the interactions which affect functions of recipient cells by mechanisms involving focal adhesion as well as extracellular matrix (ECM) interactions. Indeed, we found MSC-EVs carried 79 cell adhesion proteins (e.g., integrins, CD44 antigen) as well as miRNAs involved in ECM-receptor interaction (Additional file 1: Table S4, Fig. 2; respectively) suggesting their possible interaction with target cells including inflamed endothelial cells. Moreover, MSC-EVs contained proteins of signaling pathways linked to focal adhesion that participate in modulation of cell motility, proliferation and survival (Additional file 1: Fig. S4, Table S5). Finally, we also identified MSC-EV proteins involved in ECM-receptor interactions (Additional file 1: Fig. S5)*.*

Global miRNA profiling by qRT-PCR showed several miRNAs to be present in MSC-EVs corresponding to the parental MSC content, as reported previously [27]. Interestingly, ten (10) miRNAs were detected in MSC-EVs only, but they were not detected in MSCs (Additional file 1: Fig. S6) that may suggest that they are under detection limit in parental cells. The analysis of miRNA types enriched in MSC-EVs (with fold change ≥ 2 compared to MSCs; conducted by miRPath tool) revealed their possible involvement in TGF-β signaling (e.g. miR-276a-3p, 761, 369-3p, 344d-3p), signaling regulating pluripotency of stem cells (miR-344d-3p, 187-3p, 376a-3p) or ECM-receptor interactions (miR-325-3p, 202-3p) (Fig. 3B). Moreover, miRNA-382-5p, 132-3p and 126-3p that contributed to pro-regenerative processes [28–30] were also detected in MSC-EVs (Additional file 1: Fig. S6).

Together, these data show that MSC-EVs are enriched in MSC-derived bioactive molecules including proteins and miRNA that may regulate phenotypic change and function of other target cells including endothelial and immune cells under normal or inflamed state.

### MSC-EVs increase angiogenic potential and viability of ECs under normal and ischemic tissue conditions

**Fig. 4:**
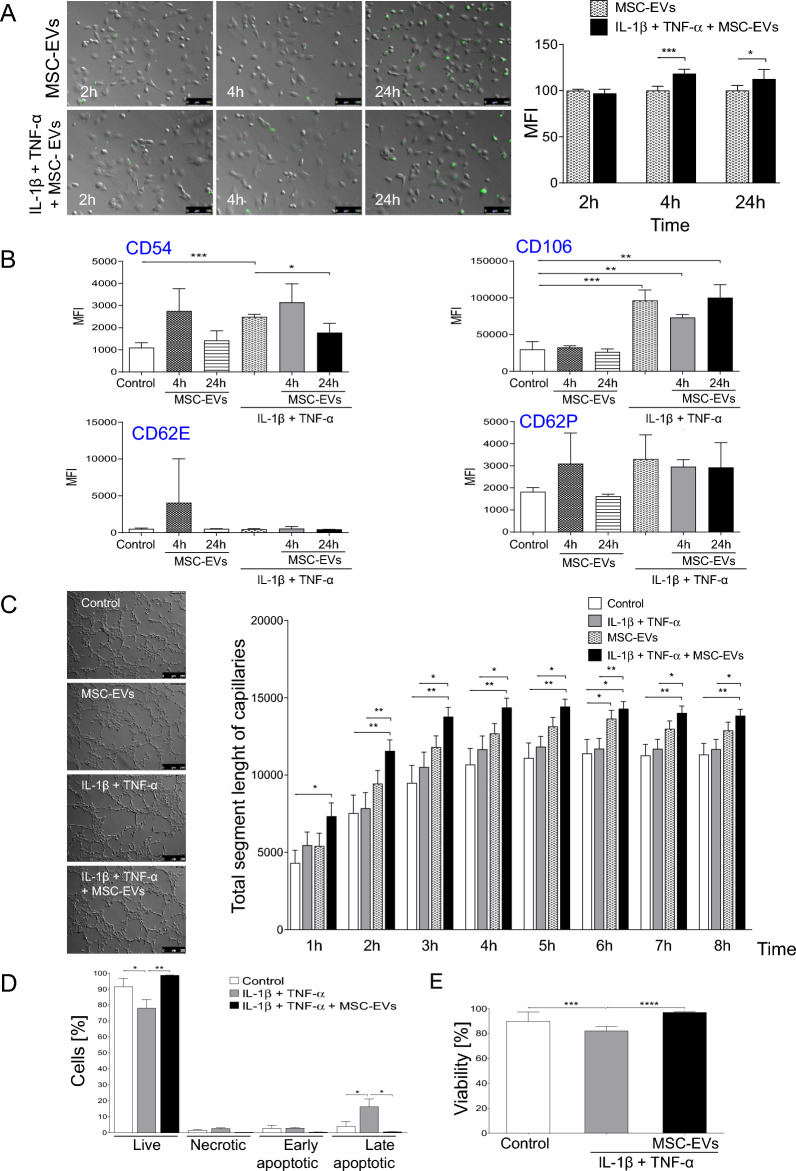
Functional effects of MSC-EVs on ECs in vitro.** A** Cellular up-take of MSC-EVs by untreated (control) or IL-1β and TNF-α pre-incubated MCECs by fluorescent microscopy. MSC-EVs were fluorescently labelled with DIO (green) and incubated with cells for 2, 4 or 24 h. (Left) Representative images of MCECs incubated with MSC-EVs. (Right) Mean fluorescence intensity (MFI) of MCECs incubated with MSC-EVs measured at different time points and analyzed by ImageJ software. Scale bar = 100 µm. **B** Expression of selected endothelial cell activation markers (CD54, CD106, CD62E, CD62P) on MCECs: untreated cells (Control), pre-incubated with IL-1β, TNF-α and/or treated with MSC-EVs for 4 h or 24 h, measured by flow cytometry. **C** (Left) Proangiogenic potential of MCECs: untreated (control), IL-1β and TNF-α-treated, MSC-EV-treated or IL-1β, TNF-α and MSC-EV-treated MCECs on Matrigel matrix. Scale bar represents 250 µm. (Right) Quantitative analysis of pro-angiogenic potential of MCECs presented as total segment length of capillaries per microscopic field at different time points (from 1 h until 8 h). **D** Viability of MCECs treated or untreated with MSC-EVs and cultured in microenvironment mimicking ischemic injury conditions by flow cytometry. MCECs were cultured in the presence of IL-1β and TNF-α under hypoxic conditions (2% O_2_). Percent content of alive (AnnV^−^/PI^−^), necrotic (AnnV^−^/PI^+^), early apoptotic (AnnV^+^/PI^−^) and late apoptotic (AnnV^+^/PI^+^) cells at 4 h post MSC-EVs were shown. **E** Viability of MCECs stained with DAPI by flow cytometry. Student *t*-test, comparison with untreated control cells (*P < 0.05, **P < 0.01, ***P < 0.001, ****P < 0.0001)

In our in vitro studies, we found that under conditions mimicking ischemic tissue (where pro-inflammatory factors and hypoxic conditions were present), the internalization of MSC-EVs by mouse vascular endothelial cells—MCECs was significantly higher when compared those obtained under control conditions, which was especially observed shortly after incubation (after 4 h) (Fig. 4A). Interestingly, in the same time, no significant change in the expression of selected adhesion molecules such as e.g. CD62E or CD62P, was observed on the surface of endothelial cells in normal and tissue injury-mimicking conditions, but the density of CD54 and especially CD106 molecule (interacting with β1 integrin containing ligand) were significantly increased on the MSC-EVs (Fig. 4B) and induced a time-dependent increase of capillary formation by endothelial cells in both control and ischemic tissue injury-mimicking conditions (Fig. 4C). Interestingly, no impact of pro-inflammatory factors on angiogenic potential of MCEC was observed when compared to control (Fig. 4C). However, we found that tissue injured conditions mimicked with inflammatory cytokines, IL-1β and TNF-α significantly reduced endothelial cell viability by increasing the late phase of apoptosis, which was reversed when MSC-EVs were added to the cells (Fig. 4D).

Thus, we found that under adverse conditions of injured and inflamed tissue, the internalization of MSC-EVs by endothelial cells was significantly enhanced allowing the molecular transfer of the EV cargo and MSC-EVs increased angiogenic activity of these cells and their survival by inhibiting apoptosis (Fig. 4A–D). The enhanced EV internalization in such conditions may be dependent on the interaction of CD106 (VCAM-1) molecule with β1 integrin (CD29) representing common MSC marker, also present on fraction of MSC-EVs (Fig. 1D).

### MSC-EVs increase capillary-forming capacity of lymphatic ECs via VEGF3 signaling

As reported above, global proteomic analysis revealed that MSC-EVs carry proteins such as Itgα5 involved in inflammatory lymphangiogenesis and neuropilin-1 (NRP1), which act as a key modulator of vascular, lymphatic, and inflammatory cell responses [25, 26].

**Fig. 5:**
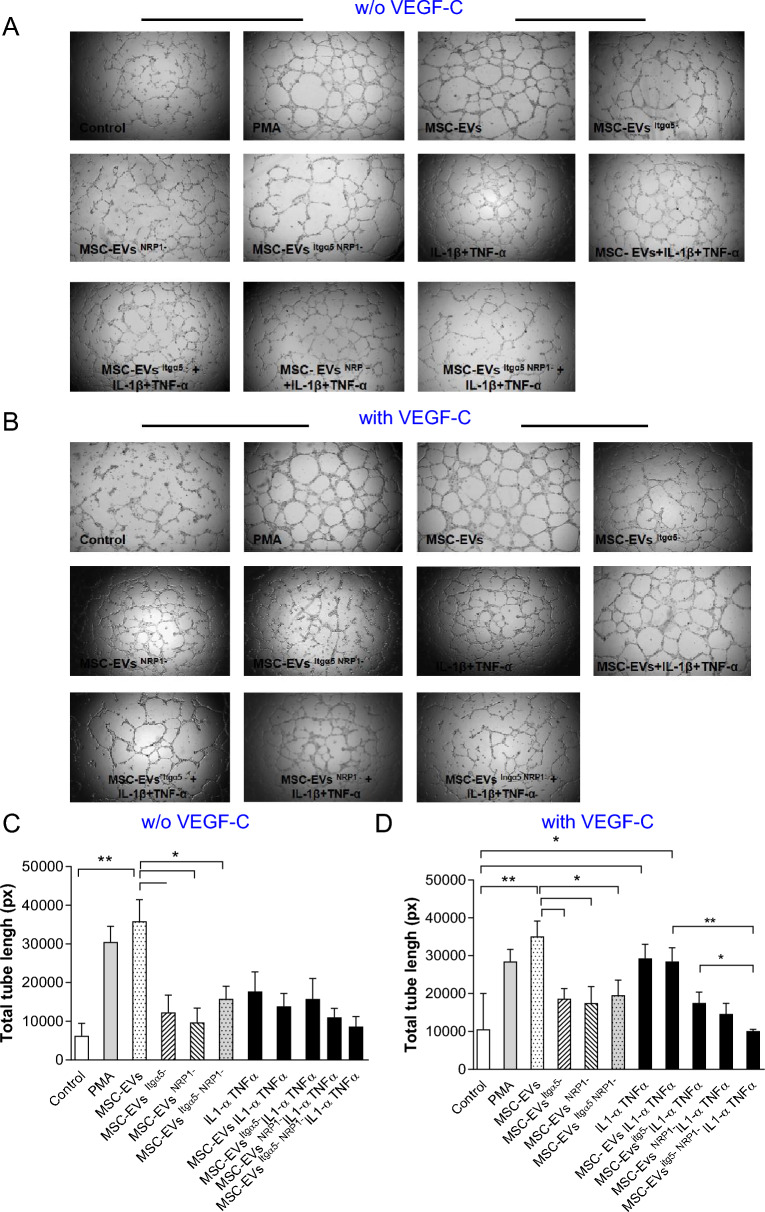
Effects of MSC-EVs on lymphatic ECs in vitro*.* Representative phase contrast photographs of LECs incubated **A** or unincubated with VEGF-C **B** and with/without MSC-EVs: native/unmodified (MSC-EVs), EVs pre-incubated with NRP1 antibody (MSC-EVs^NRP1−^) or EVs pre-incubated with Itgα5 antibody (MSC-EVs^Itgα5−^). LECs treated with PMA represent positive control. Bar graph represent the quantitative analysis of total tube length formed by LECs in absence **C** and in the presence **D** of VEGF-C. Student *t*-test, comparison with untreated control cells (*P < 0.05, **P < 0.01)

We found that under normal conditions, MSC-EVs increased capillary length of lymphatic endothelial cells (LEC) and this in absence or in presence of VEGF-C (Fig. 5). Interestingly, neutralizing the interaction with blocking specific antibody of either Itgα5 (MSC-EVs^Itgα5−^) or NRP1 (MSC-EVs^NRP1−^) alone or in combination (MSC-EVs^Itgα5−/NRP1−^) prevented the ability of MSC-EVs to increase the capillary length structure both in absence and in presence of VEGF-C (Fig. 5).

In the absence of VEGF-C and in inflammatory conditions, none of the MSC-EVs had an effect on capillary length of LEC. However, in the presence of VEGF-C, inflammatory conditions significantly increased total tube length whereas MSC-EVs were not able to induce a further increase in capillary length (Fig. 5B, D). Blockade either of Itgα5 or NRP1 alone, using MSC-EVs^Itgα5−^ or MSC-EVs^NRP1−^ significantly reduced the ability of inflammatory conditions to increase LEC tube formation in the presence of VEGF-C (Fig. 5B, D). Interestingly, concomitant blockade of Itgα5 or NRP1 with MSC-EVs^Itgα5−/NRP1−^ completely abrogated the effect of inflammatory conditions in presence of VEGF-C (Fig. 5B, D).

Collectively, we demonstrate for the first time a mandatory role of VEGF3 signaling and Itgα5 and NRP1 carried by MSC-EVs in regulating lymphangiogenesis depending on lymphatic ECs.

### MSC-EVs exhibit retention into ischemic tissues in vivo

**Fig. 6:**
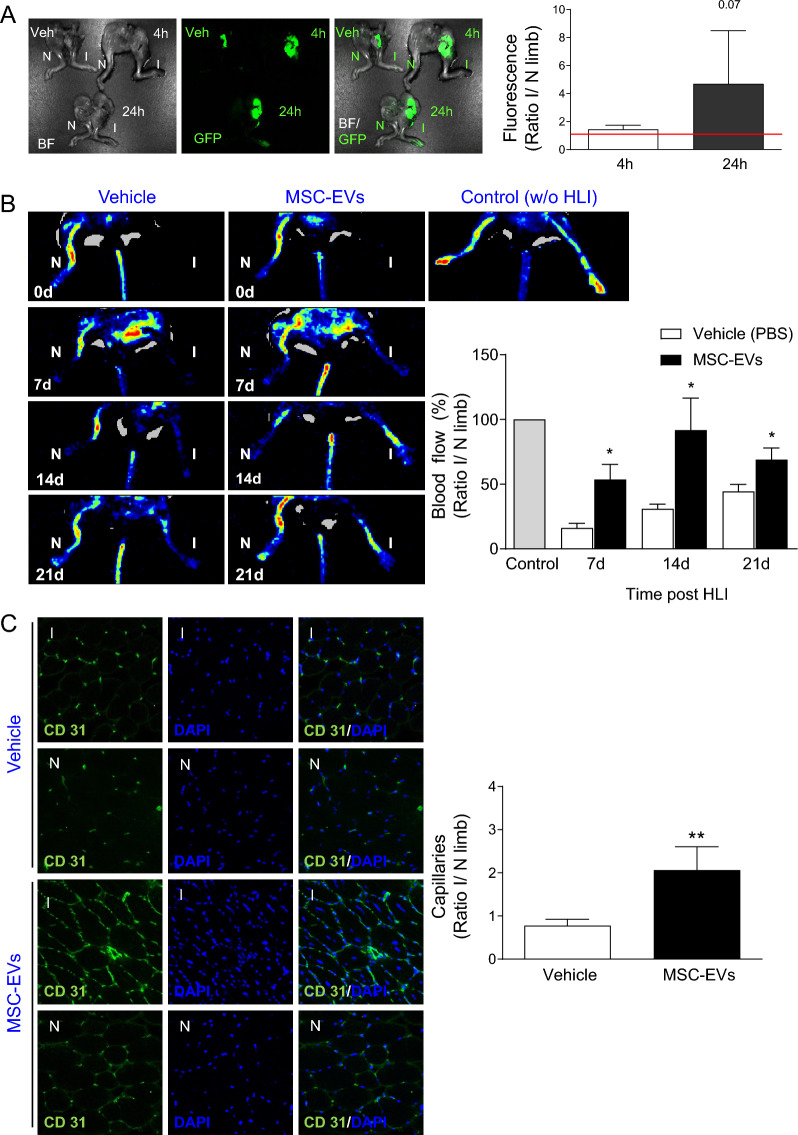
Bio-distribution and regenerative potential of systematically delivered MSC-EVs in murine model of hindlimb ischemia (HLI) in vivo. **A** (Left) Representative images of non-ischemic (N) and ischemic (I) limbs at 4 or 24 h after *i.v.* administration of GFP-positive MSC-EVs (green) or vehicle (PBS) by MAESTRO In-Vivo fluorescence imaging system. (Right) Average fluorescence emitted from limbs—presented as a ratio of fluorescence in ischemic (I) to non-ischemic (N) limb. Student *t*-test, comparison with animals at 4 h after EV administration. Red line represent signal of untretaed animals. **B** (Left) Representative images of blood flow measured prior limb ischemia injury (Control, w/o HLI) and at day 7, 14 and 21 after HLI and EV administration by Laser Doppler. (Right) Quantification of blood flow presented as a ratio of tissue reperfusion in ischemic (I) to perfusion in healthy non-ischemic (N) limb. Student *t*-test, comparison to vehicle (PBS)-treated animals (*P < 0.05). **C** Analysis of capillary density in muscle tissues at 21d post HLI with/without EV treatment. (Left) Representative images of CD31 (green) stained capillaries. Ischemic (I) and non-ischemic (N) tissue sections were co-stained with DAPI (blue) to visualize nuclei. (Right) Quantification of total number of the stained capillaries per microscopic field in skeletal muscles of vehicle- or MSC-EV-treated mice post mortem. Data are presented as ratio of total number of capillaries in ischemic (I) to non-ischemic (N) limb. Mann–Whitney test, comparison to vehicle (PBS)-treated animals (**P < 0.01)

Next, the in vivo biodistribution study of MSC-EVs were conducted following *i.v.* injection, in order to verify whether the EVs exhibit tropism into the site of ischemic injury. For such purpose, C57Bl/6J mice underwent hindlimb ischemia injury and were *i.v.* injected with GFP-positive MSC-EVs (100 µg) to investigate biodistribution of MSC-EVs in the plasma and selected organs at 4 or 24 h post administration. At 4 h, MSC-EVs were mostly distributed to the kidneys with lower level in the liver, spleen and lungs (Additional file 1: Fig. S7A, B). Twenty-four hours’ post-injection, the lower fluorescence signal was detected in liver, kidneys and lungs when compared to the level observed at 4 h post-injection. Importantly, we found increased localization of MSC-EVs in ischemic limbs at 24 h post-injection, when compared to control untreated limb, indicating enhanced retention of the EVs in injured inflamed and hypoxic tissues (Fig. 6A).

### MSC-EVs enhance recovery of blood flow and vasculature in ischemic tissues in vivo

To evaluate pro-regenerative potential of MSC-EVs in murine model of HLI, mice underwent hindlimb ischemia injury by ligating-excising the left FA and were further *i.v.* injected either with MSC-EVs or vehicle (PBS) as a control every 3 days up to 21 days (Additional file 1: Fig. S1). Tissue blood perfusion was measured before FA occlusion (baseline) as well as at day 7, 14 or 21 post HLI, by laser Doppler perfusion imaging technique (Fig. 6B). Importantly, we found significant improvement in blood tissue perfusion in ischemic limb following MSC-EV administration at day 7, 14 and 21 after HLI injury, when compared to the vehicle-treated mice (Fig. 6B). We found also greater number of capillaries in ischemic skeletal muscle tissue harvested form animals treated with MSC-EVs when compared to the vehicle-treated mice, as indicated by immunohistochemical staining against the endothelial cell marker CD31 (Fig. 6C).

Taken together, these results demonstrate pro-angiogenic potential of MSC-EVs in ischemic tissue in vivo, which was associated with increased capillary densities leading to improve blood tissue perfusion in vivo.

### MSC-EVs enhance NO production and endothelial NO synthase (eNOS) activation in ischemic limbs

**Fig. 7:**
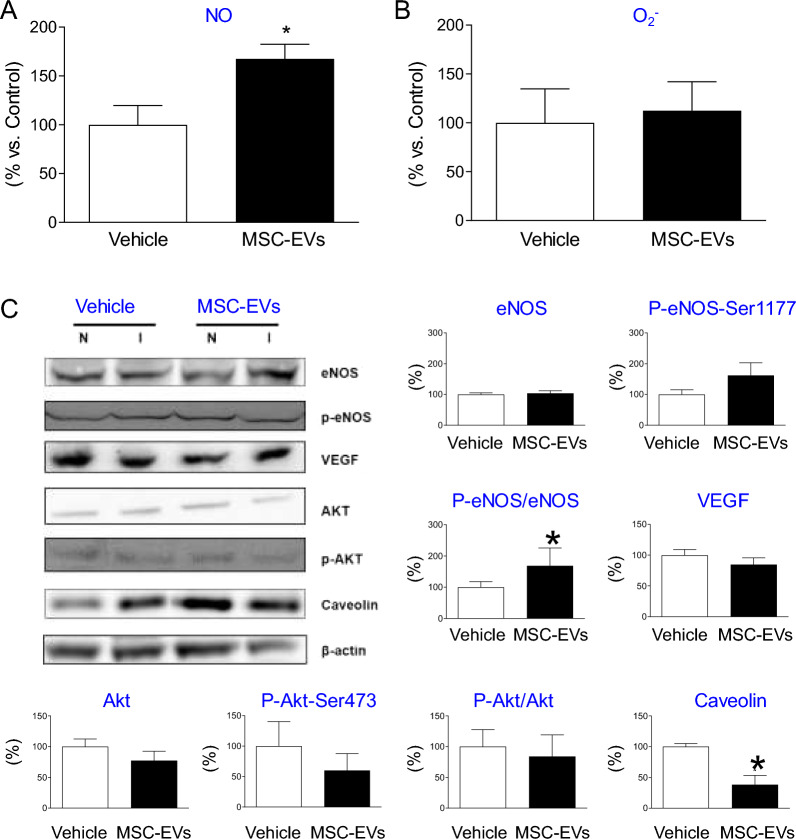
Activation of angiogenesis related pathways in skeletal muscles after MSC-EV treatment in vivo. Quantification of NO **A** and O_2_^−^ **B** production in skeletal muscles by EPR. **C** (Left) Representative Western immunoblotting analysis of protein levels of eNOS, VEGF, Akt and their phosphorylated variants, caveolin and β-actin proteins in muscle tissues from ischemic (I) and non-ischemic (N) limb. (Right/Bottom) Quantitative analysis of expression and phosphorylation of eNOS, VEGF, Akt and caveolin-1. Values are presented as a ratio of ischemic (I) to non-ischemic (N) signal amplitude/protein expression and calculated as a percent (%) of signal in control tissues form vehicle (PBS)-treated animals. Mann–Whitney test, comparison to vehicle-treated samples (*P < 0.05)

The production of reactive oxygen species (O_2_^−^) and nitric oxide (NO) in skeletal muscle following the HLI and MSC-EV administration were analyzed by EPR technique. We found significantly enhanced production of NO, without modification of O_2_^−^, in the ischemic skeletal muscles in MSC-EV-treated mice at 21 days post HLI, when compared to vehicle-treated mice (Fig. 7A, B).

Western blotting analysis revealed that MSC-EV treatment enhanced phosphorylation of NO producing enzyme: eNOS on its activator site (Ser 1177) and decreased caveolin-1 expression showing that increased NO production was due to the activation of eNOS pathway. In contrast, neither expression of VEGF and Akt nor activation of Akt was influenced by MSC-EV treatment. In comparison, in non-ischemic tissues such as aorta we did not observe any increase in NO production or eNOS and VEGF expressions. The level of phosphorylated form of Akt was lower after MSC-EV treatment when compared to vehicle-treated group (Additional file 1: Fig. S8).

Taken together, MSC-EV treatment stimulates NO production in ischemic tissues, which may be involved in NO-mediated angiogenesis in injured tissues.

### MSC-EVs promote lymphangiogenesis in ischemic tissues in vivo

**Fig. 8:**
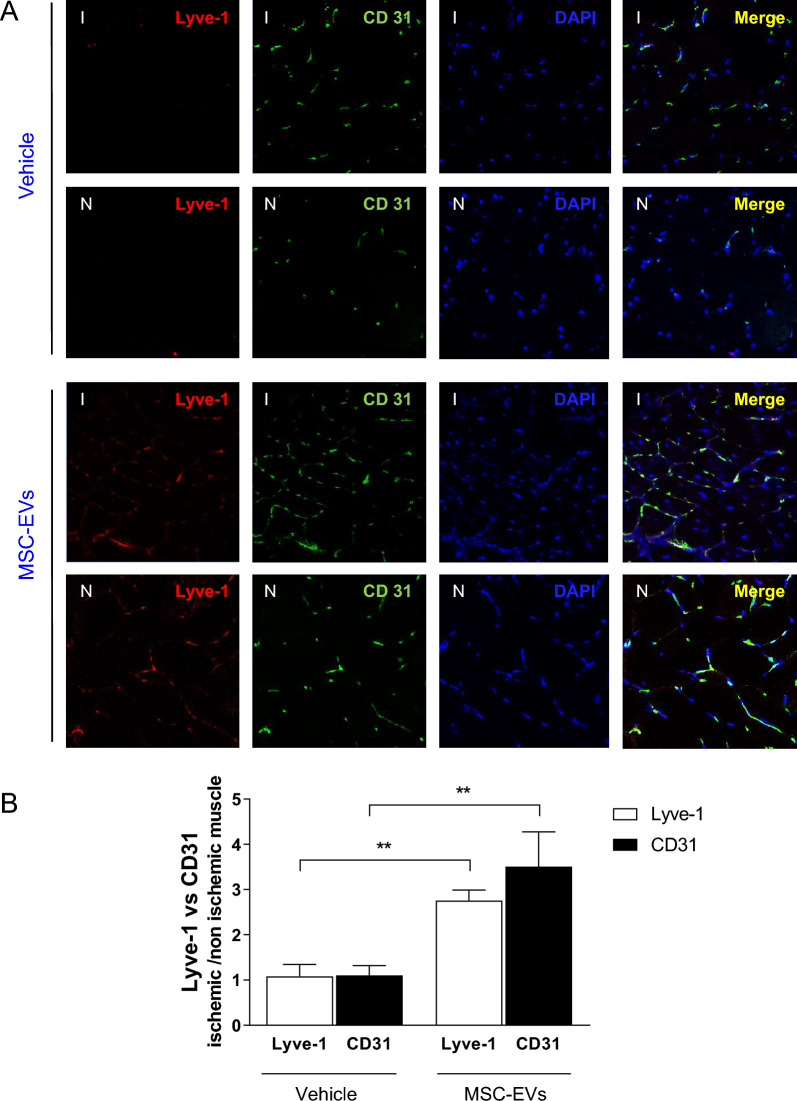
Lymphangiogenesis in ischemic tissues after treatment with MSC-EVs in vivo*.* **A** Representative immunofluorescence images of gastrocnemius muscle sections harvested from ischemic (I) and non-ischemic limb (N) of vehicle- or MSC-EV-treated mice at 21 days post HLI. **B** Quantification of Lyve 1 and CD31-stained vessels in tissues of vehicle- or MSC-EV-treated animals. Data are expressed as ratio of ischemic (I) to non-ischemic (N). Student *t*-test, comparison to vehicle-treated samples (**P < 0.01)

Immunohistochemical staining of skeletal muscle tissue sections for the endothelial cells (CD31) and lymphatic endothelial (Lyve) markers were analyzed by confocal microscopy. Importantly, we observed increased number of not only CD31, but also Lyve1 expressing cells in ischemic muscle sections following MSC-EV treatment, when compared to vehicle-treated animals (Fig. 8). As shown in Fig. 8A, many lymphatic and blood vessels coexisted in intimate contact, indeed no significant overlap was noted between Lyve1 and CD31 staining. These data demonstrate that MSC-EVs not only induce angiogenesis, but also lymphangiogenesis in injured tissues in vivo.

## Discussion

In the present study, we have described the pro-angiogenic and pro-lymphangiogenic roles of MSC-EVs on ECs under ischemic conditions, which may play their role via the transfer of bioactive cargo consisting of various microRNAs and proteins including Itgα5 and NRP1. Although MSCs secrete various soluble active factors [31], we focused exclusively on MSC-EVs that were isolated using a sequential centrifugation method including double ultracentrifugation step.

Importantly for the functional effects of MSC-EVs in tissue repair, our proteomic data revealed the presence in MSC-EVs of proteins involved in many biological pathways including those involved in tissue repair and angiogenesis. We found that MSC-EVs were enriched in proteins involved the regulation of several metabolic processes including glycolysis, cellular protein catabolism, receptor-mediated endocytosis, cell–matrix and cell–cell adhesion, cell migration as well as pro-regenerative processes such as wound healing, angiogenesis and their positive regulation. Among such proteins, we identified Itgα5, which is known to be involved in inflammatory lymphangiogenesis [25] and NRP1, which acts as a key modulator of vascular, lymphatic, and inflammatory cell responses [26]. Furthermore, microRNA profiling showed that MSC-EVs are enriched in miRNAs involved in TGF-β signaling, regulation of stem cells pluripotency, focal adhesion signaling and ECM-receptor interactions. Thus, we found that MSC-EVs are enriched in MSC-derived bioactive molecules such as proteins and miRNA that can regulate phenotype, function and homing of target cells including endothelial and immune cells under normal or inflamed state.

Indeed, we found that MSC-EVs enhanced angiogenic properties and viability of capillary ECs under normal and inflammatory/ischemic conditions via the inhibition of the late phase of apoptosis. Importantly, we report here for the first time that MSC-EVs enhanced capillary-formation capacity of lymphatic ECs via regulation of the VEGFR3 signaling pathway with a mandatory role of Itgα5 and NRP1 under normal and “tissue injured” conditions. Thus, in our multiple in vitro assays, we demonstrated that MSC-EVs induced both angiogenesis and lymphangiogenesis, which are essential for the improvement of organ function after injury. Importantly, in mouse model of ischemic hind limb injury, we found greater recovery of blood flow in MSC-EVs-treated mice when compared to controls, which was associated with an increase in NO production in ischemic muscles. Indeed, EVs increased eNOS phosphorylation and decreased caveolin-1 expression in ischemic muscles.

Bio-distribution analysis of EVs revealed increased staining of MSC-EVs-GFP in the ischemic hind limb compared to non-ischemic muscles. Interestingly, dual CD31 and Lyve1 immunostaining in muscle cryosections, indicating angiogenesis and lymphangiogenesis, respectively, showed that MSC-EVs enhanced both processes in ischemic tissues in vivo*.*

Thus, the present study provides repertoire of MSC-EV proteins and miRNA bioactive factors to meet therapeutic needs to rescue organ ischemic injury via the enhancement of both angiogenesis and lymphangiogenesis processes. These findings also highlight Itgα5 and NRP1 carried by MSC-EVs as potential targets to promote lymphangiogenesis.

The functional effects of EVs depend on several factors including their origin and harvesting. The method used in this study to isolate MSC-EVs by differential centrifugation was performed in accordance with the recommendations of the MISEV position and allowed us to isolate mixed population of small and medium/large EVs from MSCs [15]. Deun et al*.* confirmed relatively high efficiency of EV isolation by differential centrifugation, which correlated with relatively low contamination with other proteins [32]. Furthermore, the isolation was performed using MSCs-derived conditioned media free of exogenous serum-derived EVs in accordance with the approach recommended by many investigators [33, 34]. Thus, MSC-EVs obtained under these experimental conditions allow in depth analysis the content and biological potential a whole population of BM-derived MSC-EVs with a good assessment of vesicle purity [33, 34]. The size distribution of murine MSC-EVs ranged from 70 to 430 nm and their antigenic profile are similar to that of human umbilical cord-derived MSC-EVs [35]. Using the Apogee A50 system followed by image flow cytometry, we confirmed expression of parental cell-specific antigens (CD29, CD44, CD90 and Sca-1) and tetraspanin (CD81), representing 1st category of proteins present in all EVs [15] as well as receptor 2 for VEGF (CD309) on their surface. Western blotting revealed expression of syntenin (representing 2nd category of proteins present in all EVs [15]) and lack of calnexin. Subsquently, high-throughput global proteomic analysis revealed presence of 82 proteins from the 100 most identified small EV markers published in ExoCarta database [36], confirming the enrichment in small EVs as well as the presence of other 1st and 2nd category proteins present in all EVs [15]. Balkom et al*.* compared proteomic profile of MSC-EVs (≤ 200 nm) published by 10 research groups and defined the following MSC-EVs hallmark proteins: COL1A1, COL1A2, LRP1, ACTN1, ALDOC, ANPEP, COL6A1, COL6A2, COL6A3, FLNB, FLNC, HBB, Thy1, VAT1, VIM [37]. Interestingly, 14 of the 15 proteins mentioned above were detected in MSC-EV samples obtained during current study.

Importantly, bioinformatic analysis of LC–MS/MS data and microarray analysis underscored that MSC-EVs contain a repertoire of proteins involved in wide range of signaling pathways involved in pro-regenerative processes such as angiogenesis. In addition, MSC-EVs were enriched with proteins involved in several biological processes, including glycolysis, receptor-mediated endocytosis, cell–matrix and cell–cell adhesion, cell migration and wound healing. Interestingly, a robust association of network proteins involved in both focal adhesion and ECM interactions was established. Taken together, in-depth proteomic analysis revealed that MSC-EVs carry proteins with significant roles in the regulation of multiple cellular processes including those associated with pro-regenerative events. Global miRNA profiling highlighted the enrichment of MSC-EVs in 10 miRNAs involved in TGF-β signaling, Hippo signaling, signaling regulating stem cell pluripotency or ECM-receptor interaction. For example, miRNA-382-5p upregulation is associated with increased proliferation, migration and tube formation of vascular endothelial cells [28], miRNA-132-3p overexpression is associated with increased antioxidant stress and anti-apoptotic ability of cardiomyoblast cells [29], whereas transplantation of miRNA-126-overexpressing MSCs improves angiogenesis in the infarcted area and cardiac function of mice [30]. Together, these data show that MSC-EVs are enriched with bioactive molecules (proteins, miRNA) that regulate phenotype, function and homing of target cells including endothelial cells.

The formation of new blood capillaries is an important component of pathological tissue repair in response to ischemia. The development of an effective collateralization in the ischemic zone involves angiogenesis, which is the sprouting of new capillaries, and arteriogenesis, the development of arterial structures from small preexisting collateral vessels. In addition, the lymphatic vasculature is essential for maintaining tissue fluid homeostasis, immune cell trafficking and nutritional lipid uptake. Therapeutic stimulation of cardiac lymphangiogenesis after myocardial infarction results in accelerated resolution of myocardial edema and inflammation, thereby promoting cardiac recovery [38]. We found that MSC-EVs increased capillary-like tube formation and protected the viability of MCECs under normal and inflammatory conditions via the inhibition of the late phase of apoptosis.

Importantly, we also found that MSC-EVs enhanced the capillary formation capacity of lymphatic ECs in normal or “injured tissue environment”. Interestingly, neutralizing the interaction of Itgα5 and NRP1 using blocking antibodies prevented the ability of MSC-EVs to increase capillary-like structures. Thus, the interaction of Itgα5 and NRP1 carried by MSC-EVs with a cell‐surface receptor and/or their internalization by LECs is essential for lymphangiogenesis. Both Itgα5 and Nrp1 participate in VEGFR-3-mediated cell signaling independent of VEGFC stimulation. Indeed, the VEGFR3/VEGFR2/NRP1 complex is known to be involved in VEGFR3 signaling to the lymphatic endothelium by controlling AKT activation [39]. Likewise, VEGFR-3 selectively associates with integrin Itgα5 to promote the growth of LECs through the activation of phosphatidylinositol 3 kinase/Akt signaling pathway [40]. Thus, we report an obligatory role of Itgα5 and NRP1 carried by MCS-EVs in stimulating lymphangiogenesis under normal and injured tissue-mimicking conditions as a potential mechanism for tissue repair. Future studies using knockout of the indicated factors would be interesting to further evaluate this phenomenon.

Finally, we provide in vivo evidence that MSC-EV treatment induces an increased homing of these EVs to ischemic tissues and stimulates blood flow recovery in ischemic limbs after femoral artery ligation. Intravenously administered MSC-EVs also showed retention in liver, kidneys, lung and spleen which is consistent with observations made by other investigators [41]. EVs remain in the circulation for a short time [41] and their blood concentration is determined by a balance between abundant secretion and rapid clearance [42]. The observed potential of MSC-EVs for retention in injured tissues may create new applications for cell-free drugs and, importantly this phenomenon may contribute to an efficient therapeutic outcome [43]. Interestingly, other stem cell-derived EVs have been shown to accumulate at sites of injury and confer substantial protection [44], similarly to the observations in the current study.

We found that MSC-EV treatment promoted the production of NO in the ischemic area, which is an important proangiogenic factor. This was associated with increased phosphorylation of eNOS on the activator site and decreased expression of caveolin-1, which acts as negative regulator of eNOS activity and NO production. On the other hand, the molecular content of MSC-EVs may on the other hand suggest that MSC-EVs may also promote angiogenesis by transferring their protein repertoire to target cells such as EGF, FGF, PDGF and VEGF as well as miRNA molecules including miRNA-382-5p, miRNA-132-3p and miRNA-126. Regarding lymphangiogenesis, to the best of our knowledge, we describe for the first time, to our knowledge, that MSC-EV treatment increased Lyve1 expression in ischemic skeletal muscle. As shown in our in vitro study, we found an obligatory role of Itgα5 and NRP1 carried by MSC-EVs in the process of capillary formation by lymphatic ECs under normal and injured tissue-mimicking conditions. We hypothesize that MSC-EVs may improve ischemic limb recovery by limiting both edema and inflammation through regulation of NRP1/or Itgα5/VEGFR-3 signaling, which requires further studies.

## Conclusions

The present study provides evidence for pro-angiogenic and pro-lymphangiogenic properties of MSC-EVs, which may be exploited for ischemic tissue repair. The results highlight a role of protein and miRNA content of MSC-EVs, including Itgα5 and NRP1, in the regulation of such processes and indicate them as potential targets to promote lymphangiogenesis in injuries and pathologies associated with peripheral ischemia.

The beneficial effect of the application of MSC-EVs in a murine model of hindlimb ischemia provides a potentially interesting therapeutic perspective for patients suffering from ischemic diseases to induce therapeutic angiogenesis and lymphangiogenesis. Based on promising preclinical data regarding the pro-regenerative efficacy and clinical safety of MSC-EVs [43], they may be an interesting future therapeutic product for cell-free therapy of ischemic tissues in human patients. However, further investigations would be required for a deeper understanding of the differential in effects of MSC-EVs on the processes of angiogenesis and lymphangiogenesis.

### Supplementary Information

**Additional file 1.** Extended material and methods, additional tables and figures.

## Data Availability

The underlying data for this article can be found in the article and its Additional file.

## Abbreviations

EVs: Extracellular vesicles
MSCs: Mesenchymal stem/stromal cells
BM: Bone marrow
MSC-EVs: Mesenchymal stem/stromal cell-derived extracellular vesicles
ECs: Endothelial cells
MCECs: Mouse cardiac endothelial cells
LECs: Lymphatic endothelial cells
HLI: Hind limb ischemia
WT: Wild type
ECM: Extracellular matrix
Itgα5: Integrin α5
NRP1: Neuropilin-1
