# Supplementary material for: Mesenchymal stem cell-derived extracellular vesicles exert pro-angiogenic and pro-lymphangiogenic effects in ischemic tissues by transferring various microRNAs and proteins including ITGa5 and NRP1
Source: J Nanobiotechnology. 2024 Feb 12;22:60. doi: 10.1186/s12951-024-02304-y (PMC10863128; doi:10.1186/s12951-024-02304-y)
Supplement: Supplementary file 1 — Additional file 1. Extended material and methods, additional tables and figures. [file 12951_2024_2304_MOESM1_ESM.pdf]

Mesenchymal stem cell-derived extracellular vesicles exert pro-angiogenic and pro-lymphangiogenic effects in ischemic tissues by transferring various microRNAs and proteins including ITGa5 and NRP1

Anna Łabędź-Masłowska[1], Luisa Vergori[2*], Sylwia Kędracka-Krok[3], Elżbieta Karnas[1], Sylwia Bobis-Wozowicz[1], Małgorzata Sekuła-Stryjewska[4], Michał Sarna[5], Ramaroson Andriantsitohaina[6#] and Ewa K. Zuba-Surma[1#]*

[1]Jagiellonian University, Faculty of Biochemistry Biophysics and Biotechnology, Department of Cell Biology, Krakow, Poland.

[2]INSERM U1063, Oxidative Stress and Metabolic Pathologies, Angers University, Angers, France.

[3]Jagiellonian University, Faculty of Biochemistry, Biophysics and Biotechnology, Department of Physical Biochemistry, Krakow, Poland.

[4]Jagiellonian University, Malopolska Centre of Biotechnology, Laboratory of Stem Cell Biotechnology, Krakow, Poland.

[5]Jagiellonian University, Faculty of Biochemistry, Biophysics and Biotechnology, Department of Biophysics, Krakow, Poland.

[6]PhyMedExp, INSERM U1046 - UMR CNRS 9214 - Université de Montpellier, Montpellier, France.

[*]These authors contributed equally to this work as first co-authors

[#]These authors contributed equally to this study and share corresponding authorship

Correspondence:

Ramaroson Andriantsitohaina, PharmD, PhD

PhyMedExp, INSERM U1046 - UMR CNRS 9214 - Université de Montpellier, Montpellier, France, E-mail : ramaroson.andriantsitohaina@inserm.fr

and

Ewa K. Zuba-Surma, PhD, DSc

Department of Cell Biology, Faculty of Biochemistry, Biophysics and Biotechnology, Jagiellonian University, Krakow, Poland, E-mail: ewa.zuba-surma@uj.edu.pl

ADDITIONAL MATERIALS AND METHODS

The detailed extended description of Materials and Methods is presented below.

Animals

Two strains of four-to-eight week old mice were used in this study: 1) C57Bl/6 wild type strain (WT) and 2) transgenic eGFP-expressing mice (C57Bl/6-Tg(GFP)J). All procedures including murine tissue isolations were performed at the Jagiellonian University (JU) in Krakow were conducted in accordance with the approval of the Ethical Committee on Animal Testing at the JU (approval number: 183/2012). Mice were supplied by Charles River (Wilmington MA, USA) and were subsequently held in the Animal Facility at the Faculty of Biochemistry, Biophysics and Biotechnology JU, for 7-day quarantine prior to their experimental use. For conducting a regenerative and bio-distribution study *in vivo*, six-to-eight week-old male C57Bl/6 mice were used. The experiments related to tissue regenerative and bio-distribution *in vivo* were conducted at the Université d'Angers in Angers, France in agreement with the guidelines and authorization with French Ministry of Agriculture regulations based on European Community standards on the care and use of laboratory animals. C57Bl/6 mice were supplied for experiments by Charles River Laboratories (L'Arbresle, France) and held in Animal Facility at Faculty of Medicine, Université d'Angers in Angers, France.

Cell culture

Isolation and culture of murine BM- derived MSCs

Murine bone marrow (BM) tissue has been used as a starting material for isolation of MSCs. BM-derived cells were harvested from tibias and femurs by flushing the bone cavities with Dulbecco's Modified Eagle's Medium/Nutrient Mixture F-12 Ham (DMEM/F12; #D8437, Merck). BM-derived cells were centrifuged (350x*g*, 6 min, RT), re-suspended in complete culture medium - DMEM/F12 supplemented with 10% fetal bovine serum (FBS; #F2442,

Merck), 100 U/ml of penicillin and 100 µg/ml of streptomycin (P/S; Gibco, ThermoFisher Scientific) and seeded into tissue culture flasks (BD Falcon, Corning). Flushed bones were fragmented and enzymatically digested with mixture of collagenase type I and II (each at a concentration of 1 mg/ml; Merck) for 1.5h at 37°C. Collagenases were inactivated by adding DMEM/F12 supplemented with 10% FBS and cells were subsequently centrifuged (350x*g*, 6 min, RT), re-suspended in DMEM/F12 with 10% FBS and transferred to culture flasks containing previously flushed BM-derived cells. Cells were cultured in a humidified atmosphere at 5% CO_2 and 37˚C for 72h, and then non-adherent cells were removed. The culture medium was changed three times a week and the passage was conducted with 0.25% trypsin/ EDTA (Gibco, ThermoFisher Scientific) when confluence of cells reached app. 90%. Cells with highest viability (>98%) were subjected for further experiments including EV harvest.

<u>Culture of MCECs</u>

Mouse Cardiac Endothelial Cells (MCECs) were purchased from CELLutions Biosystems Inc. (#CLU510, Ontario, Canada) and cultured in Dulbecco's Modified Eagle's Medium (DMEM; #D5796, Merck) supplemented with 10 mmol/L HEPES (HyClone, GE Healthcare Life Sciences) and 5% FBS (#F2442, Merck) on 0.1% gelatin coated cell culture dishes (BD Falcon, Corning). The cells were passaged with Tryple Select Enzyme 1X (ThermoFisher Scientific) after reaching a confluence of app. 80-90%. The cells were cultured in a humidified atmosphere of 5% CO_2 at 37°C.

<u>Culture of LECs</u>

Mouse Primary Dermal Lymphatic Endothelial Cells (LECs) derived from C57BL/6 mouse strain were purchased from Cell Biologics (#C57-6064L, Chicago, USA) and cultured in tissue culture flasks pre-coated with gelatin-based coating solution (#6950, Cell Biologics) in dedicated Culture Complete Mouse Endothelial Cell Medium (#M1168, Cell Biologics).

Culture medium was changed three times a week, and the cells were passaged after reaching a confluence of app. 80%. The cells were cultured in a humidified atmosphere of 5% CO_2 at 37°C.

Isolation of EVs

Murine MSC-derived extracellular vesicles (MSC-EVs) were isolated from cell culture supernatants of BM-derived MSCs using a sequential centrifugation method including double ultracentrifugation step. MSCs at passages 4, 5 or 6 were used for EVs harvest. Briefly, MSCs reaching a confluence of 80% were washed twice with PBS (w/o Ca^{2+}, Mg^{2+}, HyClone) and further cultured in ultracentrifuged medium DMEM/F12 containing 10% FBS in order to eliminate EVs and other small particles of FBS origin. The used medium was ultracentrifuged at 100,000x*g* for 18h, at 4°C using Optima XPN-90 ultracentrifuge with type 50.2 Ti fixed-angle rotor (Beckman Coulter). MSCs were subsequently cultured for 48h at 37°C in a humidified atmosphere of 5% CO_2. The following features of parental MSCs were examined prior to harvesting of MSC-conditioned media for further EV isolation: i) potential signs of infection of cell culture (correct result: orange or red-raspberry clear culture medium); ii) cell morphology (correct result: features characterizing MSCs - spindle-shaped, elongated cells possessing fibroblast-like morphology); iii) presence of detached cells (qualitative assessment; correct result: lack of detached cells in culture) by light microscopy. The cell-conditioned media were collected only from cell culture flasks positively fulfilled the listed above quality control parameters. The viability of MSCs was greater than 98% as it was assessed by a manual dye-exclusion method following staining with 0.4% Trypan Blue solution (ThermoFisher Scientific).

For MSC-EV isolation, the collected cell-conditioned media were centrifuged at 350x*g* for 10 min in 4°C to remove single detached cells. The supernatants were transferred into new tubes (Sarstedt) and then centrifuged at 500x*g* for 10 min in 4°C to remove remaining cells and cell

debris. Subsequently, the supernatants were immediately processed or frozen at -80°C. In the next step, to remove smaller cellular debris and apoptotic bodies, the fresh or frozen/refrozen cell-conditioned media were subsequently centrifuged at 2,000x*g* for 20 min at 4°C. EVs (and large protein/ RNA complexes) were finally collected by ultracentrifugation of supernatants in PP Oak Ridge Tubes (Thermo Fisher) at 100,000x*g* for 75 min at 4°C (in a T-865 fixed angle rotor, Thermo Fisher; placed in Optima XPB ultracentrifuge, Beckman Coulter), washed once in ultrapure PBS (#BE17-512F, Lonza) and pelleted (centrifuged at 100,000x*g* for 75 min at 4°C using the rotor and centrifuge listed above). Freshly isolated MSC-EVs were resuspended in PBS (#BE17-512F, Lonza) and transferred to Protein LoBind Tubes (Eppendorf). The protein content of MSC-EVs samples was quantified using the Bradford reagent assay and a standard curve developed based on a series of BSA protein concentration (ranging from 0.0 to 2.0 mg/ml; Merck). All absorbance determinations were made using a Multiscan FC Microplate Reader (Thermo Fisher).

Particle size analysis

Tunable resistive pulse sensing (TRPS, qNano, Izon) was used to analyze the size distribution of nanoparticles in MSC-EV samples. The particle measurement platform was set up and calibrated using CPC200 beads (Izon Science) according to manufacturer's instructions. Prior to analysis, MSC-EV samples were diluted in ultrapure PBS (#BE17-512F, Lonza) and passed through a 0.45 μm Acrodisc Minispike syringe filter (Sigma-Aldrich) to remove aggregates. MSC-EVs were measured using a NP200 nanopore (analysis range 85–500 nm; Izon Science) with 20 or 10 mbar pressure. Stretch and voltage were set up in order to achieve a stable higher than 100 nA current. Samples were analyzed for 5 min or until 1000 particles were counted. Data processing and analysis were carried out on the Izon Control Suite software v2.2 (Izon Science).

Analysis of EV morphology

To analyze morphology of MSC-EVs accordingly to the recommendations published by the International Society for Extracellular Vesicles (ISEV)[1], Atomic Force Microscopy (AFM) technique was used. For AFM analysis, a drop of MSC-EVs sample (containing 5-10 μg of proteins) was placed on a freshly cleaved mica surface and incubated for 20 min at RT and then gently washed with PBS to remove any unattached MSC-EVs. Images of the topography of surface containing attached MSC-EVs were taken using PeakForce Tapping (PFT) mode. The use of PFT allowed to directly monitor the tip-sample interaction, due to the fact that the mode is based on measuring force-displacement curves (it allows for control of the force exerted on the analyzed sample, which is crucial when investigating delicate samples).

Flow cytometric analysis of EV antigenic profile

Surface markers on MSC-EVs were directly analyzed using a high resolution flow cytometry. The used Apogee A50-Micro Flow Cytometer (Apogee Flow Systems, UK) was equipped with 488 nm (blue) and 633 nm (red) lasers. The following fluorochrome- conjugated antibodies against murine antigens were used according to the manufacturers' protocols: CD29-APC (clone: HMβ1-1, Biolegend), CD44-APC (clone: IM7, Biolegend), CD81-APC (clone: Eat2, BD Bioscence), CD90-APC (clone: 30-H12, Biolegend), CD309-APC (clone: Avas12, Biolegend) and Sca-1-APC (clone: E13-161.7, Biolegend) as well as the following isotype controls: Armenian Hamster IgG-APC (clone: HTK888, Biolegend), Rat IgG2a, κ-APC (clone: RTK2758, Biolegend) and Rat IgG2b, κ-APC (clone: RTK4530, Biolegend). MSC-EVs were in the same time stained with SYTO RNA Select dye (ThermoFisher Scientific) binding RNA molecules. Briefly, 2 μg of MSC-EVs were placed to 1.5 ml protein low-binding tubes in PBS and 250 μl of staining solutions containing 0.7-1.0 μg of particular antibody as well as 5 nM SYTO RNA Select dye (depleted of aggregates) were added to the EV samples. MSC-EVs suspensions were incubated with antibodies for 30 min in the dark at $4^{o}C$ and immediately

analyzed by Apogee A50-Micro flow cytometer. The obtained results were analyzed using Apogee Histogram software (Apogee Flow Systems).

In order to confirm the presence of the indicated antigens on MSC-EVS, imaging cytometer ImageStream X Mark II (Merck) was additionally used to visualize the stained MSC-EVs.

Western blotting

To analyze the presence of EV specific markers in the isolated MSC-EV specimens Western blotting analysis was performed. Briefly, MSC-EV samples were resuspended in non-reducing sample buffer, separated by 12.5% SDS-PAGE and transferred to a PVDF membrane (Merck). Membrane was blocked in PBS containing 5% (w/v) nonfat dry milk powder and 0.1% Tween-20 and subsequently immunolabeled with rabbit anti-mouse CD9 (1:1000, AntibodyGenie), goat anti-mouse calnexin (1:500, ThermoFisher Scientific), rabbit anti-mouse syntenin (1:1000, ThermoFisher Scientific) and murine anti-mouse β-actin (1:2000, ThermoFisher Scientific) antibody. Next, horseradish peroxidase-conjugated goat anti-rabbit antibody (1:4000, Dako), rabbit anti-goat antibody (1:4000, ThermoFisher Scientific) or goat anti-mouse antibody (1:4000, ThermoFisher Scientific) were incubated with PVDF membranes for 1h. Detection of products was performed using Supersignal West Dura chemiluminescent substrate (Pierce). Imaging of specific bands was performed using a ChemiDoc MP system (Bio-Rad) and Image Lab software (version 5.1, Bio-Rad).

Global proteomic analysis of EV content

Initial preparation of protein samples

MSC-EVs pellet were lysed in lysing buffer composed of 4% SDS (Fluka Chemie), 0.1 M DTT (Merck), 0.1M Tris (Roche) in Tris-HCl (BioShop) at pH 7.6 and sonicated two times for 5 min (320W, 30s on/off), using Bioruptor UCD-200 homogenizer (Diagenode) for total protein harvesting. The samples were further incubated at 95°C for 5 min and centrifuged

at 16,000x*g* for 15 min at 20°C. The supernatant was collected and stored at -80°C until further preparation.

Filter Aided Sample Preparation for LC-MS/MS analysis

MSC-EVs samples were prepared for global proteomic analysis by liquid chromatography-tandem mass spectrometry (LC-MS/MS) using the filter assisted sample preparation (FASP) method described by *Wisniewski et al.* [2]. Briefly, 100 µg of protein lysate was diluted in 300 µl of 8M urea (Lab Empire) in 50 mM ammonium bicarbonate (ABC, Sigma) and mixed with freshly added DTT (final concentration was 50mM) by vortexing for 15 min. Subsequently, the samples were centrifuged (21,000x*g*, 15 min, 20°C) and applied on the 30 kDa cut-off filter (Vivacon 500, Sartorius Stedim Biotech GmbH). The next steps including alkylation, washing with the urea solution and washing with 50 mM ABC were performed according to the Protocol 1 derived from FASP Protein Digestion Kit Use and Storage Instructions (Expendeon). After washing step, 1 µg of Lys-C protease (Promega) resuspended in ABC solution was added. After on-filter protein digestion (overnight, 37°C) the resulting peptides were spin down (14,000x*g*, 15 min, 20°C) into a new tubes and further washed out of the membrane two times with ABC solution (14,000x*g*, 15 min, 20°C). Then, 1 µg of trypsin (Promega) resuspended in ABC solution was added to the column. After overnight digestion at 37°C, the obtained peptides were centrifuged (14,000x*g*, 15 min, 20°C) to a new tubes and further washed out of the membrane according to the procedure conducted after Lys-C digestion. As a result, there were two peptide mixtures for each sample: 1) peptides after digestion with Lys-C protease and 2) peptides after digestion with trypsin.

Fractionation of peptides

The obtained peptides were fractionated according to the protocol described by *Wisniewski et al.*[3] *via* strong anion exchange (SAX) using tip columns packed with six layers of resin disks (Empore Anion-SR, 47 mm Extraction Disks, Supelco, Sigma-Aldrich). Peptides obtained after

Lys-C digestion were separated into four fractions using BRUB buffers (Britton & Robinson universal buffer) at pH of 11, 6, 4 and 2, respectively, while peptides obtained after trypsin digestion were separated into two fractions with BRUB buffers at pH 5 and 2. Then, the obtained fractions underwent purification using tip columns packed with six layers of C18 resin disks (Empore Octadecyl C18, 47 mm Extraction Disks, Supelco, Sigma-Aldrich) according to the following protocol: precondition with methanol (JT Baker); washing with 60% acetonitrile (ACN, JT Baker)/1% acetic acid (JT Baker) solution; equilibration with 1% acetic acid; sample loading; washing with 1% acetic acid; elution with 60% ACN/1% acetic acid solution. Then, the samples were vacuum-dried and further suspended in 2% ACN/ 0.05% trifluoroacetic acid (TFA, JT Baker) solution for LC-MS/MS analysis.

LC-MS/MS acquisition

The MSC-EV-derived peptides were analysed by mass spectrometry (MS) using high-resolution Q-Exactive mass spectrometer (Thermo Scientific) coupled with an UltiMate 3000RS LC nanoSystem (Thermo Scientific). The peptides were loaded onto a C18 precolumn (Acclaim PepMap Nano trap Column, Thermo Scientific; ID 75 µm, length 20 mm, particle size 3 µm, pore size 100Å) using 2% ACN with 0.05% TFA as a mobile phase at a flow rate of 5 µm/min and then separated on C18 analytical column (Acclaim PepMap RLSC C18, ThermoScientific; ID 75 µm, length 500 mm, particle size 2 µm, pore size 100Å) using a 4h gradient of ACN from 2% to 40% in 0.05% formic acid (JT Baker). Finally, peptides were ionized in a Digital PicoView 550 ion source (New Objective, Woburn, MA, USA). Top 12 method was used for mass spectrometry measurement with full MS and MS/MS resolution of 70,000 and 17,500, respectively.

LC-MS/MS data analysis

In the conducted experiment 3 samples (biological replicates) of MSC-EVs were profiled. The data obtained from LC-MS/MS were analyzed using Proteome Discoverer 1.4 (Thermo

Scientific). Briefly, an inhouse MASCOT server (v. 2.5.1, Matrix Science, Boston, MA, USA) was used for searching against the Swissprot_201509 database, which was restricted to *Mus musculus* taxonomy. The parameters of searching were as follows: enzyme - trypsin; number of missed cleavages - 2; static modification - carbamidomethylation (C); dynamic modifications - oxidation (M); peptide mass tolerance - 10 ppm and fragment mass tolerance - 20 mmu. The data from six fractions of a given sample were searched together. Validation of search result was performed in Proteome Discoverer 1.4 using Percolator algorithm according to *Kall et al.* [4]. False discovery rate (FDR) was set below 1%.

The total number of identified proteins was greater than 2200. Only proteins identified at least in 2 out of the 3 MSC-EV samples based on 2 or more unique peptides for each protein, were considered for further analyses. For the proteins identified in MSC-EVs, GO enrichment analysis were performed in DAVID Functional Annotation Bioinformatics Microarray Analysis (https://david.ncifcrf.gov/ home.jsp). In the conducted analysis was used *p* value threshold of 0.05. Moreover, proteins identified in MSC-EVs was also analyzed for their affiliation to specific signaling pathways based on the GO: Panther Pathways annotation, taking FDR below 5% or 1%. In the next stage, for the proteins identified in the MSC-EVs, an interaction analysis was carried out using the commercially available application STRING Ver. 10.5 (https://string-db.org/). Moreover, ExoCarta data base [5] was used to confirm or exclude presence of 100 top proteins often identified in small EVs (previously called exosomes).

Analysis of microRNA content in EVs

To determine the microRNA content of MSC-EVs real-time qRT-PCR profiling was used. Briefly, total RNA (containing small RNAs) was purified from MSC-EV specimens using miRCURY RNA Isolation Kit - Cell & Plant (Exiqon) according to the manufacturer's protocol. Samples were treated with Turbo DNAse (according to the manufacturer's instruction; Ambion, ThermoFisher Scientific) to ensure complete removal of genomic DNA contamination. The

purity and RNA concentration were determined using a Nano Photometer (Implen). Only RNA preps with the concentration above 5 ng/µl and the purity about 2.00 as measured by absorbance ratio $A_{260/280}$, were used for further analysis. Next, cDNA synthesis was conducted using the Universal cDNA Synthesis Kit II (Exiqon) according to the manufacturer's instructions in C1000 Touch Thermal Cycler (Bio-Rad). The cDNA products were subsequently diluted and transferred to the Ready-to-use Mouse and Rat microRNA (miRNA) PCR Panel I + II, V3.M (Exiqon) and quantified using Power SYBR Green Master Mix (Applied Biosystems, ThermoFisher Scientific) based real-time qPCR and Locked Nucleic Acids (LNA) enhanced miRNA specific primers. The qPCRs were run on the QS6 Real-Time PCR System (ThermoFisher Scientific) using the reaction parameters recommended by Exiqon. Data were analyzed using GeneEx software (bioMCC, Freising, Germany) and further by mirPath v.3 (DIANA-Lab, University of Thessaly, Greece) dedicated to the assessment of miRNA regulatory roles and the identification of controlled pathways as described by *Vlachos et al*. [6]. Three (3) independent samples (biological replicates) of MSC-EVs were analyzed in this study. For the most enriched miRNAs in MSC-EVs (fold change in expression ≥ 2.0 when compared to parental MSCs), the heat-map showing their involvement in functional pathways was generated with DIANA mirPath web tool.

Internalization of EVs by ECs *in vitro*

<u>Fluorescent Labeling of MSC-EVs</u>

To examine MSC-EV uptake by target cells such as vascular endothelial cells *in vitro,* MSC-EVs samples were stained with the Vibrant DiO Cell-Labeling Solution lipophilic dye (ThermoFisher Scientific) and additionally with SYTO RNA Select green fluorescent cell stain (ThermoFisher Scientific) according to the manufacturers' protocols - to label membranes lipids as well as RNA inside the vesicles, respectively. MSC-EVs were stained for 20 min in the dark at RT and freshly used for experiments.

Analysis of cellular uptake of MSC-EVs

MCEC cells were seeded on glass bottom dishes (Ø 35 mm, WillCo Wells B.V.) at a density of $5x10^4$ cells/dish in complete DMEM medium (#D5796, Merck) supplemented with 5% FBS (#F2442, Merck). Cells were washed twice with PBS (w/o Ca^{2+}, Mg^{2+}, HyClone) after 24h post seeding, and divided into two groups:

1) Control – further MCECs culture in complete DMEM medium with 5% FBS for 24h at 37^oC in humidified atmosphere containing 5% CO_2 and 21% O_2;

2) Microenvironment mimicking inflammation and hypoxia – further MCECs culture in complete DMEM medium with 1% FBS, 10 ng/ml IL-1β (#211-11B, Peprotech) and 10 ng/ml TNF-α (#315-01, Peprotech) for 24h at 37^oC in humidified atmosphere containing 5% CO_2 and 2% O_2.

Next, fluorescent labelled MSC-EVs (50 µg) were added into each dish and MCEC cells were further incubated at 37^oC in humidified atmosphere containing 5% CO_2 and 21% O_2 (Control) or 5% CO_2 and 2% O_2 (Microenvironment of inflammation and hypoxia). Internalization of MSC-EVs by MCEC cells was examined after 2, 4 and 24h of incubation with EVs by using Leica DMI6000B inverted fluorescent microscope (ver. AF7000, Leica Microsystems) equipped with Leica Application Suite X Software (Leica Microsystems). Mean fluorescence intensity of MCEC were measured using ImageJ software (Maryland, USA).

Capillary-like tube formation assay *in vitro*

Assessment of pro-angiogenic potential of MCECs

To investigate an impact of MSC-EVs on the pro-angiogenic potential of vascular endothelial cells - MCECs, capillary formation was assessed *in vitro.* Briefly, 24-well plates (BD Falcon, Corning) were coated with Matrigel Matrix Grow Factor Reduced (100µl/well, Corning) and incubated at 37^oC for 30 min. MCECs were seeded at a density of $5x10^4$ cells/well in the EBM-

2 Basal Medium supplemented with EGM-2MV SingleQuots Kit (both from Clonetics, Lonza). The seeded MCECs were divided into four following subgroups:

1) Control – further incubation of MCECs in EBM-2 supplemented with EGM-2MV SingleQuots Kit;
2) Microenvironment of inflammation and hypoxia - further incubation of MCECs in EBM-2 supplemented with EGM-2MV SingleQuots Kit, 10 ng/ml IL-1β (#211-11B, Peprotech) and 10 ng/ml TNF-α (#315-01, Peprotech);
3) Control + MSC-EVs – further incubation of MCECs in EBM-2 supplemented with EGM-2MV SingleQuots Kit and 10 μg/ml MSC-EVs;
4) Microenvironment of inflammation and hypoxia + MSC-EVs - further incubation of MCECs in EBM-2 supplemented with EGM-2MV SingleQuots Kit, 10 ng/ml IL-1β (#211-11B, Peprotech), 10 ng/ml TNF-α (#315-01, Peprotech) and 10 μg/ml MSC-EVs.

Capillary-like structure formation by MCECs was investigated and recorded at 37°C in a 5% CO_2 chamber (PeCon GmbH, Erbach, Germany) mounted on a Leica DMI6000B inverted microscope equipped with a dry 10x, NA 0.25 objective, integrated modulation contrast optics and a digital DFC360FX CCD camera (Leica Microsystems). The capillary-like tube formation of MCECs was time-lapse recorded for 8h using Leica Application Suite X software (Leica Microsystems). The total tube length was quantified using the Image J software.

<u>Assessment of pro-lymphangiogenic potential of LECs</u>

The formation of three-dimensional lymphatic capillary-like structures by endothelial cells of lymphatic vessels- LECs treated with MSC-EVs was examined by performing a Matrigel-based tube formation assay. Each well of a μ-slide (ibidi GmbH) was filled with 10 μL of ECMgel (#E1270, Sigma ECMgel, Merck), which was allowed to polymerize for 30 min at 37°C. The LEC cells were seeded at a density of $1x10^4$ cells/well and cultured in endothelial medium in the presence or absence of 100ng/ml VEGF C (#**SRP4634,** Merck). The LECs were treated

with MSC-EVs and/or recombinant murine IL-1β (10 ng/ml) (#211-11B, Peprotech), recombinant murine TNF-α (10 ng/ml) (#315-01, Peprotech) to induce inflammatory conditions. In another set of experiments, mouse/rat anti-neuropilin-1 antibody (#AF566, R&D system) and anti-Integrin alpha 5 (CD49e) antibody (#103908, BioLegend) was pre-incubated with MSC-EVs for 1h at 4°C, and then centrifuged to eliminate excess of blocking antibody, before to be added to cells. A positive control was performed with 20 ng/mL PMA (#**P8139,** Merck). After 18h of incubation, the tube formation images were captured at 4-fold magnification using the Olympus CK40 (Olympus CK40, Olympus Corporation). The total tube length and number of tubes were quantified using the Image J software.

Expression of EC activation markers *in vitro*

To investigate a level of expression markers of activated vascular endothelial cells in various conditions, immunofluorescence staining and flow cytometry analyses of cells were performed. Briefly, MCEC were seeded at a density of 10^5 cells/ dish (Ø 60 mm, Falcon, Corning) in complete DMEM medium (#D5796, Merck) with 5% FBS (#F2442, Merck). Cells were washed twice with PBS (w/o Ca^{2+}, Mg^{2+}, HyClone) after 24h and divided into two groups:

1) Control – further culture of MCECs in complete DMEM with 5% FBS for 24h at 37°C in humidified atmosphere containing 5% CO_2 and 21% O_2;

2) Microenvironment mimicking inflammation and hypoxia – further culture of MCECs in DMEM with 1% FBS, 10 ng/ml IL-1β (#211-11B, Peprotech) and 10 ng/ml TNF-α (#315-01, Peprotech) for 24h at 37°C in humidified atmosphere containing 5% CO_2 and 2% O_2.

Next, into MCECs incubated in inflammation and hypoxia microenvironment following factors were added: 10 ng/ml IL-1β, 10 ng/ml TNF-α or 10 ng/ml IL-1β, 10 ng/ml TNF-α and 10 μg/ml MSC-EVs. Cell culture medium was removed after 4 or 24h of incubation and MCECs were washed twice with PBS (w/o Ca^{2+}, Mg^{2+}, HyClone), and passaged. Cells were further centrifuged (300x*g*, 5 min., RT), re-suspended in staining medium (DMEM supplemented with

1% FBS) and stained with the following fluorochrome- conjugated anti-mouse antibodies: CD54-APC (clone: YN1/1.7.4, Biolegend), CD62E-PE (clone: 10E9.6, BD Bioscence), CD62P-Alx647 (clone: RB40.34, BD Bioscence), CD106-APC (clone: 429, Biolegend) for 30 min in the dark at 4°C. Samples were next washed with PBS (w/o Ca^{2+}, Mg^{2+}, HyClone), centrifuged (300x*g*, 6 min, RT) and re-suspended in staining medium. DAPI (3 μM, ThermoFisher Scientific) was added 10 min prior to analysis to exclude dead cells from further analysis. Cells were analyzed using LSR Fortessa flow cytometer (Becton Dickinson).

Murine model of hind limb ischemia (HLI) *in vivo*

To induce unilateral hind limb ischemia (HLI), the ligation on the left femoral artery (FA) was performed according to the procedure described by *Niiyama et al.* established at the Stanford University, CA, USA [7]. Briefly, male C57BL/6 mice (Charles River Laboratories, L'Arbresle, France), six-to-eight week old were weighted and then placed into the anesthesia induction chamber containing 1–3% Aerrane Isoflurane (Baxter) in 100% oxygen at a flow rate of 1L/min. Then, the animal was placed in the supine position onto the pre-operating table and connected to a continuous flow of Aerrane Isoflurane. After removing the hair from the hindlimbs, the mouse was placed over a draped heated pad on the operating table with temperature controlling module. Using scalpel, an incision of the skin, approximately 1 cm long, were made from the knee towards the medial thigh. To expose the neurovascular bundle, the membranous femoral sheath was gently pierced. Then, using a clean set of fine forceps and cotton swab, FA was dissected and separated from the femoral vein and nerve at the proximal location near the groin. After the dissection, a strand of 6-0 silk suture (Ethicon) underneath the proximal end of FA were passed and the proximal FA were occluded using knots. Then, FA separated from the femoral vein at the distal location close to the knee and finally the vessel was occluded using knots. The segment of FA between the distal and proximal knots were

carefully transected to avoid piercing the femoral vein wall. Then, the incision was closed with 5-0 nylon suture (Ethicon).

Mice were randomly divided into two subgroups (N=8-9/group): 1) Vehicle (PBS, Control group) and 2) MSC-EVs (EV treated group). Respectively, PBS or MSC-EVs (10 µg/ml of blood) were intravenously administrated via the tail vein into animals from both groups, every 3 days for 21 days of experiment.

The total blood volume for every mouse was calculated based on consideration that the total blood volume in the mouse is in the range of 6–8 ml /100 g of body weight [8]. The calculation of MSC-EV doses were presented in Table S1.

At 7, 14 and 21 days post FA occlusion, blood flow in both limbs (healthy and ischemic) were measured as described below. At 21 days post HLI, animals were sacrificed by cervical dislocation and muscle tissues were taken for further biochemical and histological analysis. The *in vivo* experimental layout was presented on Figure S1.

Analysis of blood flow in tissues *in vivo*

In order to provide a functional evidence of ischemia as well as further blood perfusion recovery, Laser Doppler perfusion imaging technique based on dynamic light scattering in tissue was used [9]. Briefly, mice were anesthetized with Aerrane Isoflurane (Baxter) as previously described and settled on a heating plate to maintain a stable cutaneous temperature in order to minimize temperature variation throughout the experiments. Limbs perfusion was measured using a Laser Doppler flow probe (PF 408, Perimed). At least 3 flow measurements were performed per mouse. Blood flow in limbs was expressed as a ratio of left (ischemic) to right (non-ischemic) limb perfusion, as described by *Limbourg et al*. [10].

Analysis of EV bio-distribution *in vivo*

To investigate the MSC-EV retention in various organs following the *i.v.* administration animal tissues were analysed with In-Vivo Fluorescence Imaging System. Briefly, male C57BL/6 mice

underwent unilateral HLI by the ligation on the left FA as previously described. At 24h post HLI, mice were injected with 100 µg GFP-positive MSC-EVs or PBS *via* the tail vein. At 4 or 24 h post MSC-EVs or PBS injection, mice were sacrificed by cervical dislocation. Then, blood as well as liver, spleen, lungs, kidneys and limbs were isolated from every mouse. For the analysis of GFP-positive MSC-EVs bio-distribution, the multispectral imaging system MAESTRO In-Vivo Fluorescence Imaging System (*Cambridge Research & Instrumentation;* Woburn, MA, USA) was used. To isolate plasma, blood was centrifuged at 350x*g*, 7 min, RT and transferred into a new 1.5 ml tubes. The respective fluorescence of isolated organs (liver, spleen, lungs, and kidneys) as well as plasma and skeletal muscles were analyzed. All the above tissues were evaluated in their entirety. The data were analyzed with the MAESTRO In-Vivo Fluorescence Imaging System software (*Cambridge Research & Instrumentation*; Woburn, MA USA).

Immunofluorescence staining and confocal microscopy

To prepare frozen sections for semi-quantitative analysis of neovascularization in MSC-EV treated tissues, both ischemic and non-ischemic gastrocnemius muscles were dissected and embedded in Tissue-Tek O.C.T Compound (Sakura Finetek), frozen in liquid N_2 and stored at -80°C until further preparation. The frozen tissues were sectioned using Leica CM3050S cryostat (Leica Biosystems). Then, cryosections (7 µm) were fixed in 100% methanol (#**34860,** Merck) for 5 min at −20°C, and nonspecific binding sites were saturated using blocking buffer composed of 5% BSA (Euromedx) in PBS for 1,5h at RT. Next, the tissue sections were incubated with rat anti-mouse CD31 antibody (marker of endothelial cells of blood vessels; 1:100, BD Biosciences) and rabbit anti-mouse Lyve1 antibody (marker of endothelial cells of lymphatic vessels; 1:200, Acris Antibodies) overnight at 4°C. Next the sections were washed tree times with PBS and incubated (for 1h at RT) with goat anti-rat FITC-conjugated IgG (1:100, Southern Biotech) to identify blood vessels or goat anti-rabbit Alexa546 (1:500,

Interchim) secondary antibody to detected Lyve1 expression. To visualize nuclei, tissue sections were stained with DAPI (300 nM, Santa Cruz Biotechology) for 3 min at RT. After final washes, the coverslips were mounted on glass slides containing stained tissue sections. LSM 700 confocal (Zeiss) was used for the optical sectioning of the tissue. Digital image recording was performed using the Laser Sharp Software. Vessels were quantified using ImageJ software and counted in at least five randomly selected fields for each muscle section, and the mean value for each section was calculated (magnification x40).

Analysis of NO and O_2^- determination production in tissues

Detection of nitric oxide (NO) and superoxide anion (O_2^-) production in mice tissues was performed using electronic paramagnetic resonance (EPR) with Fe^{2+} diethyldithiocarbamate (DETC) as a spin trap.

At 21 days post FA ligation, animals were euthanized, then aortas and both ischemic and non-ischemic muscles were dissected and incubated in Krebs-HEPES buffer containing $Fe(DETC)_2$ solution for analysis of NO production or deferoxamin, DETC and CMH solution for analysis of O_2^- production.

Prior to EPR analysis, Krebs-HEPES buffer containing NaCl (5.786 g/L), KCl (0.35 g/L), $CaCl_2{\cdot}2H_2O$ (0.368 g/L), $MgSO_4{\cdot}7H_2O$ (0.296 g/L), K_2HPO_4 (0.14 g/L), $NaHCO_3$ (2.1 g/L), glucose (1.999 g/L), HEPES (4.766 g/L) and L-arginine (0.8 mmol/L; all reagents were purchased from Merck) were prepared. Then, in case of NO analysis, NaDETC (3.6 mg, Merck) and $FeSO_4{\cdot}7H_2O$ (2.25 mg, Merck) were dissolved under N_2 gas bubbling in two separate tubes containing 10 ml of ice-cold Krebs-HEPES buffer. After dissolving, both solutions were rapidly mixed to obtain 0.4 mM $Fe(DETC)_2$, which was used immediately to incubate freshly isolated tissues for 45 min at 37°C.

Prior to O_2^- production analysis, the ice-cold Krebs-HEPES buffer was rapidly mixed with deferoxamin (25µM, Merck), DETC (5µM, Merck) and CMH (500µM, Noxigen). The

prepared solution was used to incubate muscle tissues for 45 min at 37°C. Then, tissues for NO and O_2^- production analysis, were immediately frozen using liquid N_2. NO and O_2^- production measurement was performed using a table-top x-band spectrometer Miniscope (Magnettech, MS200, Berlin, Germany). Recording were made at 77°K using a Dewar flask. Instrument setting was as follow: 10 mW of microwave power, 1 mT of amplitude modulation frequency, 60 s of sweep time and 5 scans.

Analysis of angiogenesis-related proteins in tissues

In each experiment, the aorta and the skeletal muscles from both ischemic and non-ischemic limbs were removed and frozen in liquid N_2 to further evaluate the expression of angiogenesis-related proteins after MSC-EV treatment. Briefly, for the Western blot analyses, the tissue samples were homogenized using Polytron PRO250 homogenizer (ProScientific, Monroe, CT) and lysed in buffer containing Halt Protease Inhibitor Coctail (1X, Merck). Proteins were separated by SDS-PAGE and transferred onto PVDF membranes (Merck). Membranes were then saturated for 1h at RT in Tris buffer containing 1% Tween-20 (Merck) and 5% BSA (Merck). Then, the membranes were incubated overnight at 4°C with one of the following primary monoclonal antibodies: anti-endothelial NOS (eNOS; 1:2500, BD Biosciences), anti-caveolin-1 (1000, BD Biosciences), anti-phospho-eNOS (Ser 1177, 1:1000, Cell Signaling Technology), anti-Akt (1:1000, Cell Signaling Technology), anti-phospho-Akt (Ser 473, 1:2000, Cell Signaling Technology), and anti-VEGF (1:1000, R&D systems). To visualize protein gel loading, a polyclonal rabbit anti-actin antibody (1:5000, Merck) was used. The membranes were then washed at least three times in Tris buffer containing 0.05% Tween-20 and incubated for 1h at RT with the appropriate horseradish peroxidase (HRP)-conjugated secondary antibody (Amersham, Piscataway, NJ). The protein-antibody complexes were detected by ECL-Plus Chemiluminescence kit (Santa Cruz Biotechnology, Santa Cruz, CA) according to manufacturer's protocol using Image Reader Las-3000 (Fujifilm).

Statistical analysis

Data are represented as mean ± SD or SEM as indicated. Statistical analyses were performed by Student's *t*-test or Mann-Whitney U-tests (non-parametric) using Prism software package 8.00 or 5.00 (GraphPAD Software, San Diego, CA). $P<0.05$ was considered to be statistically significant.

ADDITIONAL TABLES

Table S1. Doses of MSC-EVs administrated (*i.v.) in vivo* (murine model of HLI).

Doses of *i.v.* administrated MSC-EVs per mouse									
No. of mouse	1	2	3	4	5	6	7	8	9
Weight [g]	20.0	22.0	21.4	22.0	22.8	24.4	20.7	23.1	24.8
Calculated blood volume [ml]	1.60	1.76	1.71	1.60	1.82	1.95	1.66	1.85	1.98
Dose of MSC-EVs [µg]/ injection	16.0	17.6	17.1	16.0	18.2	19.5	16.6	18.5	19.8

The doses of MSC-EVs were calculated for individual animals considering blood volume - based on recommendation published by *Hoff et al.*[8].

Table S2. List of top 100 proteins identified in small EVs (previously called exosomes) published in ExoCarta data base and their presence in purified MSC-EVs.

No.	*Gene symbol*	*Accesion number*	*Name*	*Presence in MSC-EVs*
1	CD9	P40240	CD9 antigen	+
2	HSPA8	P63017	Heat shock 71 kDa protein 8	+
3	PDCD6IP	Q9WU78	Programmed cell death 6-interacting protein)	-
4	GAPDH	P16858	Glyceraldehyde-3-phosphate dehydrogenase	-
5	ACTB	P60710	Actin, cytoplasmic 1	+
6	ANXA2	P07356	Annexin A2	+
7	CD63	P41731	CD63 antigen	+
8	SDCBP	O08992	Syntenin-1	+
9	ENO1	P17182	α-enolase	+
10	HSP90AA1	P07901	Heat shock protein HSP 90α	+
11	TSG101	Q61187	Tumor susceptibility gene 101 protein	+
12	PKM	P52480	Pyruvate kinase PKM	+
13	LDHA	P06151	L-lactate dehydrogenase α chain	+
14	EEF1A1	P10126	Elongation factor 1-α 1	+
15	YWHAZ	P63101	14-3-3 protein ζ/δ	+
16	PGK1	P09411	Phosphoglycerate kinase 1	+
17	EEF2	P58252	Elongation factor 2	+
18	ALDOA	P05064	Fructose-bisphosphate aldolase A	+
19	HSP90AB1	P11499	Heat shock protein HSP 90-β	+
20	ANXA5	P48036	Annexin A5	+
21	FASN	P19096	Fatty acid synthase	+
22	YWHAE	P62259	14-3-3 protein ε	+
23	CLTC	Q68FD5	Clathrin heavy chain 1	+
24	CD81	P35762	CD81 antigen	+
25	ALB	P07724	Serum albumin	+
26	VCP	Q01853	Transitional endoplasmic reticulum ATPase	+
27	TPI1	P17751	Triosephosphate isomerase	+
28	PPIA	P17742	Peptidyl-prolyl cis-trans isomerase A	+
29	MSN	P26041	Moesin	+
30	CFL1	P18760	Cofilin-1	+

31	PRDX1	P35700	Peroxiredoxin-1	+
32	PFN1	P62962	Profilin-1	+
33	RAP1B	Q99JI6	Ras-related protein Rap-1b	+
34	ITGB1	P09055	Integrin β-1	+
35	HSPA5	P20029	Endoplasmic reticulum chaperone BiP	-
36	SLC3A2	P10852	4F2 cell-surface antigen heavy chain	+
37	HIST1H4A	P62806	Histone H4	+
38	GNB2	P62880	Guanine nucleotide-binding protein subunit β-2	+
39	ATP1A1	Q8VDN2	Sodium/potassium-transporting ATPase subunit α-1	+
40	YWHAQ	P68254	14-3-3 protein ϑ	+
41	FLOT1	O08917	Flotillin-1	+
42	FLNA	Q8BTM8	Filamin-A	+
43	CLIC1	Q9Z1Q5	Chloride intracellular channel protein 1	+
44	CDC42	P60766	Cell division control protein 42 homolog	+
45	CCT2	P80314	T-complex protein 1 subunit beta	+
46	A2M	Q6GQT1	α-2-macroglobulin-P	+
47	YWHAG	P61982	14-3-3 protein γ	+
48	TUBA1B	P05213	Tubulin α-1B chain	-
49	RAC1	P63001	Ras-related C3 botulinum toxin substrate 1	+
50	LGALS3BP	Q07797	Galectin-3-binding protein	+
51	HSPA1A	Q61696	Heat shock 70 kDa protein 1A	+
52	GNAI2	P08752	Guanine nucleotide-binding protein G(i) subunit alpha-2	+
53	ANXA1	P10107	Annexin A1	+
54	RHOA	Q9QUI0	Transforming protein RhoA	-
55	MFGE8	P21956	Lactadherin	+
56	PRDX2	Q61171	Peroxiredoxin-2	-
57	GDI2	Q61598	Rab GDP dissociation inhibitor beta	+
58	EHD4	Q9EQP2	EH domain-containing protein 4	+
59	ACTN4	P57780	α-actinin-4	+
60	YWHAB	Q9CQV8	14-3-3 protein α/β	-
61	RAB7A	P51150	Ras-related protein Rab-7a	+
62	LDHB	P16125	L-lactate dehydrogenase B chain	+

63	GNAS	P63094	Guanine nucleotide-binding protein G(s) subunit alpha isoform short	-
64	TFRC	Q62351	Transferrin receptor protein 1	+
65	RAB5C	P35278	Ras-related protein Rab-5C	-
66	ARF1	P84078	ADP-ribosylation factor 1	-
67	ANXA6	P14824	Annexin A6	+
68	ANXA11	P97384	Annexin A11	+
69	ACTG1	P63260	Actin, cytoplasmic 2	-
70	KPNB1	P70168	Importin subunit β-1	+
71	EZR	P26040	Ezrin	+
72	ANXA4	P97429	Annexin A4	+
73	ACLY	Q91V92	ATP-citrate synthase	+
74	TUBA1C	P68373	Tubulin α-1C chain	-
75	RAB14	Q91V41	Ras-related protein Rab-14	+
76	HIST2H4A	P62806	Histone H4	+
77	GNB1	P62874	Guanine nucleotide-binding protein G(I)/G(S)/G(T) subunit β-1	+
78	UBA1	Q02053	Ubiquitin-like modifier-activating enzyme 1	+
79	THBS1	P35441	Thrombospondin-1	-
80	RAN	P62827	GTP-binding nuclear protein Ran	+
81	RAB5A	Q9CQD1	Ras-related protein Rab-5A	+
82	PTGFRN	Q9WV91	Prostaglandin F2 receptor negative regulator	+
83	CCT5	P80316	T-complex protein 1 subunit ε	+
84	CCT3	P80318	T-complex protein 1 subunit γ	+
85	BSG	P18572	Basigin	+
86	AHCY	P50247	Adenosylhomocysteinase	+
87	RAB5B	P61021	Ras-related protein Rab-5B	-
88	RAB1A	P62821	Ras-related protein Rab-1A	-
89	LAMP2	P17047	Lysosome-associated membrane glycoprotein 2	+
90	ITGA6	Q61739	Integrin α-6	+
91	HIST1H4B	P62806	Histone H4	+
92	GSN	P13020	Gelsolin	+
93	FN1	P11276	Fibronectin	+
94	YWHAH	P68510	14-3-3 protein η	+

95	TUBA1A	P68369	Tubulin α-1A chain	-
96	TKT	P40142	Transketolase	+
97	TCP1	P11983	T-complex protein 1 subunit α	+
98	STOM	P54116	Erythrocyte band 7 integral membrane protein	+
99	SLC16A1	P53986	Monocarboxylate transporter 1	-
100	RAB8A	P55258	Ras-related protein Rab-8A	-
Total no. of the proteins identified in MSC-EVs				**82**

List of proteins identified in MSC-EVs by LC-MS/MS were further analysed based on published list of top 100 proteins often identified in small EVs. Plus (+) means presence of selected protein whereas minus (-) absence of selected protein in MSC-EV samples.

Table S3. List of hallmark proteins identified in MSC-EVs (≤ 200 nm) according to data by *van Balkom et al.* [11].

No.	*Symbol*	*Name*	*Presence in MSC-EVs*
1	COL1A1	Collagen alpha-1(I) chain	+
2	COL1A2	Collagen alpha-2(I) chain	+
3	LRP1	Prolow-density lipoprotein receptor-related protein 1	+
4	ACTN1	Alpha-actinin-1	+
5	ALDOC	Fructose-bisphosphate aldolase C	+
6	ANPEP	Aminopeptidase N	+
7	COL6A1	Collagen alpha-1(VI) chain	+
8	COL6A2	Collagen alpha-2(VI) chain	+
9	COL6A3	Collagen alpha-3(VI) chain	-
10	FLNB	Filamin-B	+
11	FLNC	Filamentin-C	+
12	HBB	Hemoglobin subunit epsilon-Y2	+
13	THY1	CD90 antigen	+
14	VAT1	Synaptic vesicle embrane protein VAT-1 homolog	+
15	VIM	Vimentin	+
Total no. of the proteins identified in MSC-EVs			14

van Balkom et al. compared MSC-EV proteomic data published by ten (10) independent research groups and further found as common MSC-EVs markers. Plus (+) means presence of selected protein, whereas minus (-) absence of selected protein in purified MSC-EV samples.

Table S4. Cell adhesion molecules identified in MSC-EVs by LC-MS/MS followed by DAVID functional annotation bioinformatics microarray analysis.

No.	*UniProt ID*	*Gene Name*
1	CD36_MOUSE	CD36 antigen (Cd36)
2	CD44_MOUSE	CD44 antigen (Cd44)
3	CD63_MOUSE	CD63 antigen (Cd63)
4	CD9_MOUSE	CD9 antigen (Cd9)
5	EDIL3_MOUSE	EGF-like repeats and discoidin I-like domains 3 (Edil3)
6	EPHB4_MOUSE	Eph receptor B4 (Ephb4)
7	FAT3_MOUSE	FAT atypical cadherin 3 (Fat3)
8	FAT4_MOUSE	FAT atypical cadherin 4 (Fat4)
9	FREM2_MOUSE	Fras1 related extracellular matrix protein 2 (Frem2)
10	FAK2_MOUSE	PTK2 protein tyrosine kinase 2 beta (Ptk2b)
11	PTK7_MOUSE	PTK7 protein tyrosine kinase 7 (Ptk7)
12	RAC1_MOUSE	RAS-related C3 botulinum substrate 1 (Rac1)
13	WISP2_MOUSE	WNT1 inducible signaling pathway protein 2 (Wisp2)
14	ACES_MOUSE	Acetylcholinesterase (Ache)
15	ANGL3_MOUSE	Angiopoietin-like 3 (Angptl3)
16	COMP_MOUSE	Cartilage oligomeric matrix protein (Comp)
17	CTNA1_MOUSE	Catenin (cadherin associated protein), alpha 1 (Ctnna1)
18	CTNB1_MOUSE	Catenin (cadherin associated protein), beta 1 (Ctnnb1)
19	CTND1_MOUSE	Catenin (cadherin associated protein), delta 1 (Ctnnd1)
20	CO6A1_MOUSE	Collagen, type VI, alpha 1 (Col6a1)
21	CO6A2_MOUSE	Collagen, type VI, alpha 2 (Col6a2)
22	COCA1_MOUSE	Collagen, type XII, alpha 1 (Col12a1)
23	COEA1_MOUSE	Collagen, type XIV, alpha 1 (Col14a1)
24	COFA1_MOUSE	Collagen, type XV, alpha 1 (Col15a1)
25	CTGF_MOUSE	Connective tissue growth factor (Ctgf)
26	CNTN1_MOUSE	Contactin 1 (Cntn1)
27	EMIL1_MOUSE	Elastin microfibril interfacer 1 (Emilin1)
28	EMIL2_MOUSE	Elastin microfibril interfacer 2 (Emilin2)

29	EMB_MOUSE	Embigin (Emb)
30	URP2_MOUSE	Fermitin family member 3 (Fermt3)
31	SEPR_MOUSE	Fibroblast activation protein (Fap)
32	FINC_MOUSE	Fibronectin 1 (Fn1)
33	FLOT2_MOUSE	Flotillin 2 (Flot2)
34	GP1BB_MOUSE	Glycoprotein Ib, beta polypeptide (Gp1bb)
35	ALS_MOUSE	Insulin-like growth factor binding protein, acid labile subunit (Igfals)
36	ITA11_MOUSE	Integrin alpha 11 (Itga11)
37	ITA2B_MOUSE	Integrin alpha 2b (Itga2b)
38	ITA4_MOUSE	Integrin alpha 4 (Itga4)
39	ITA5_MOUSE	Integrin alpha 5 (fibronectin receptor alpha)(Itga5)
40	ITA6_MOUSE	Integrin alpha 6 (Itga6)
41	ITAL_MOUSE	Integrin alpha L (Itgal)
42	ITAM_MOUSE	Integrin alpha M (Itgam)
43	ITAV_MOUSE	Integrin alpha V(Itgav)
44	ITB1_MOUSE	Integrin beta 1 (fibronectin receptor beta)(Itgb1)
45	ITB2_MOUSE	Integrin beta 2 (Itgb2)
46	ITB3_MOUSE	Integrin beta 3 (Itgb3)
47	ITB5_MOUSE	Integrin beta 5 (Itgb5)
48	LAMB1_MOUSE	Laminin B1 (Lamb1)
49	LAMA2_MOUSE	Laminin, alpha 2 (Lama2)
50	LAMA4_MOUSE	Laminin, alpha 4 (Lama4)
51	LAMA5_MOUSE	Laminin, alpha 5 (Lama5)
52	LAMC1_MOUSE	Laminin, gamma 1 (Lamc1)
53	LG3BP_MOUSE	Lectin, galactoside-binding, soluble, 3 binding protein (Lgals3bp)
54	MFGM_MOUSE	Milk fat globule-EGF factor 8 protein (Mfge8)
55	MYH10_MOUSE	Myosin, heavy polypeptide 10, non-muscle (Myh10)
56	MYH9_MOUSE	Myosin, heavy polypeptide 9, non-muscle (Myh9)
57	NEO1_MOUSE	Neogenin (Neo1)
58	NCAM1_MOUSE	Neural cell adhesion molecule 1 (Ncam1)
59	NID1_MOUSE	Nidogen 1 (Nid1)

60	NID2_MOUSE	Nidogen 2 (Nid2)
61	OMD_MOUSE	Osteomodulin (Omd)
62	PARVB_MOUSE	Parvin, beta (Parvb)
63	POSTN_MOUSE	Periostin, osteoblast specific factor (Postn)
64	KPCD2_MOUSE	Protein kinase D2 (Prkd2)
65	PTPRF_MOUSE	Protein tyrosine phosphatase, receptor type, F (Ptprf)
66	PTPRS_MOUSE	Protein tyrosine phosphatase, receptor type, S (Ptprs)
67	RELN_MOUSE	Reelin (Reln)
68	OSTP_MOUSE	Secreted phosphoprotein 1 (Spp1)
69	SN_MOUSE	Sialic acid binding Ig-like lectin 1, sialoadhesin (Siglec1)
70	SVEP1_MOUSE	Sushi, von Willebrand factor type A, EGF and pentraxin domain containing 1 (Svep1)
71	TLN1_MOUSE	Talin 1 (Tln1)
72	TENA_MOUSE	Tenascin C (Tnc)
73	TSP1_MOUSE	Thrombospondin 1 (Thbs1)
74	TSP2_MOUSE	Thrombospondin 2 (Thbs2)
75	TSP4_MOUSE	Thrombospondin 4 (Thbs4)
76	THY1_MOUSE	Thymus cell antigen 1, theta (Thy1)
77	BGH3_MOUSE	Transforming growth factor, beta induced (Tgfbi)
78	TINAL_MOUSE	Tubulointerstitial nephritis antigen-like 1 (Tinagl1)
79	VINC_MOUSE	Vinculin (Vcl)

The analysis was conducted for proteins which were identified in MSC-EVs on the basis at least 2 unique peptides. According to GenomeNet application, cell adhesion molecules family consisting of integrins, immunoglobulins, selectins and cadherins, may play a critical role in a wide array of biological processes such as *e.g.* maintaining of homeostasis, inflammation, the immune response or embryogenesis.

Table S5. <u>Focal adhesion molecules</u> identified in MSC-EVs by LC-MS/MS followed by DAVID functional annotation bioinformatics microarray analysis.

No.	*UniProt_ID*	*Gene Name*
1	RAP1B_MOUSE	RAS related protein 1b (Rap1b)
2	RAC1_MOUSE	RAS-related C3 botulinum substrate 1 (Rac1)
3	RAC2_MOUSE	RAS-related C3 botulinum substrate 2 (Rac2)
4	RAP1A_MOUSE	RAS-related protein-1a (Rap1a)
5	ROCK2_MOUSE	Rho-associated coiled-coil containing protein kinase 2 (Rock2)
6	ACTB_MOUSE	Actin, beta (Actb)
7	ACTN4_MOUSE	Actinin alpha 4 (Actn4)
8	ACTN1_MOUSE	Actinin, alpha 1 (Actn1)
9	CAN2_MOUSE	Calpain 2 (Capn2)
10	COMP_MOUSE	Cartilage oligomeric matrix protein (Comp)
11	CTNB1_MOUSE	Catenin (cadherin associated protein), beta 1 (Ctnnb1)
12	CDC42_MOUSE	Cell division cycle 42 (Cdc42)
13	CO1A1_MOUSE	Collagen, type I, alpha 1 (Col1a1)
14	CO1A2_MOUSE	Collagen, type I, alpha 2 (Col1a2)
15	CO3A1_MOUSE	Collagen, type III, alpha 1 (Col3a1)
16	CO4A1_MOUSE	Collagen, type IV, alpha 1 (Col4a1)
17	CO5A2_MOUSE	Collagen, type V, alpha 2 (Col5a2)
18	CO6A1_MOUSE	Collagen, type VI, alpha 1 (Col6a1)
19	CO6A2_MOUSE	Collagen, type VI, alpha 2 (Col6a2)
20	DIAP1_MOUSE	Diaphanous related formin 1 (Diaph1)
21	FINC_MOUSE	Fibronectin 1 (Fn1)
22	FLNC_MOUSE	Filamin C, gamma (Flnc)
23	FLNA_MOUSE	Filamin, alpha (Flna)
24	FLNB_MOUSE	Filamin, beta (Flnb)
25	GRB2_MOUSE	Growth factor receptor bound protein 2 (Grb2)
26	ITA11_MOUSE	Integrin alpha 11 (Itga11)
27	ITA2B_MOUSE	Integrin alpha 2b (Itga2b)
28	ITA4_MOUSE	Integrin alpha 4 (Itga4)

29	ITA5_MOUSE	Integrin alpha 5 (fibronectin receptor alpha) (Itga5)
30	ITA6_MOUSE	Integrin alpha 6 (Itga6)
31	ITAV_MOUSE	Integrin alpha V (Itgav)
32	ITB1_MOUSE	Integrin beta 1 (fibronectin receptor beta) (Itgb1)
33	ITB3_MOUSE	Integrin beta 3 (Itgb3)
34	ITB5_MOUSE	Integrin beta 5 (Itgb5)
35	ILK_MOUSE	Integrin linked kinase (Ilk)
36	LAMB1_MOUSE	Laminin B1 (Lamb1)
37	LAMA2_MOUSE	Laminin, alpha 2 (Lama2)
38	LAMA4_MOUSE	Laminin, alpha 4 (Lama4)
39	LAMA5_MOUSE	Laminin, alpha 5 (Lama5)
40	LAMC1_MOUSE	Laminin, gamma 1 (Lamc1)
41	MK01_MOUSE	Mitogen-activated protein kinase 1 (Mapk1)
42	MK03_MOUSE	Mitogen-activated protein kinase 3 (Mapk3)
43	MP2K1_MOUSE	Mitogen-activated protein kinase kinase 1 (Map2k1)
44	ML12B_MOUSE	Myosin, light chain 12B, regulatory (Myl12b)
45	PARVB_MOUSE	Parvin, beta (Parvb)
46	PK3CG_MOUSE	Phosphoinositide-3-kinase, catalytic, gamma polypeptide (Pik3cg)
47	PGFRB_MOUSE	Platelet derived growth factor receptor, beta polypeptide (Pdgfrb)
48	PDGFC_MOUSE	Platelet-derived growth factor, C polypeptide (Pdgfc)
49	KPCB_MOUSE	Protein kinase C, beta (Prkcb)
50	PP1A_MOUSE	Protein phosphatase 1, catalytic subunit, alpha isoform (Ppp1ca)
51	RELN_MOUSE	Reelin (Reln)
52	OSTP_MOUSE	Secreted phosphoprotein 1 (Spp1)
53	TLN1_MOUSE	Talin 1 (Tln1)
54	TENA_MOUSE	Tenascin C (Tnc)
55	TSP1_MOUSE	Thrombospondin 1 (Thbs1)
56	TSP2_MOUSE	Thrombospondin 2 (Thbs2)
57	TSP4_MOUSE	Thrombospondin 4 (Thbs4)
58	VASP_MOUSE	Vasodilator-stimulated phosphoprotein (Vasp)
59	VINC_MOUSE	Vinculin (Vcl)

The analysis were conducted exclusively for proteins which were identified in MSC-EVs on the basis at least 2 unique peptides. “Focal adhesion” term defines cell-to-matrix adhesion, which play essential role in the following biological processes: cell motility, proliferation, differentiation, survival or gene regulation.

ADDITIONAL FIGURES

Figure S1

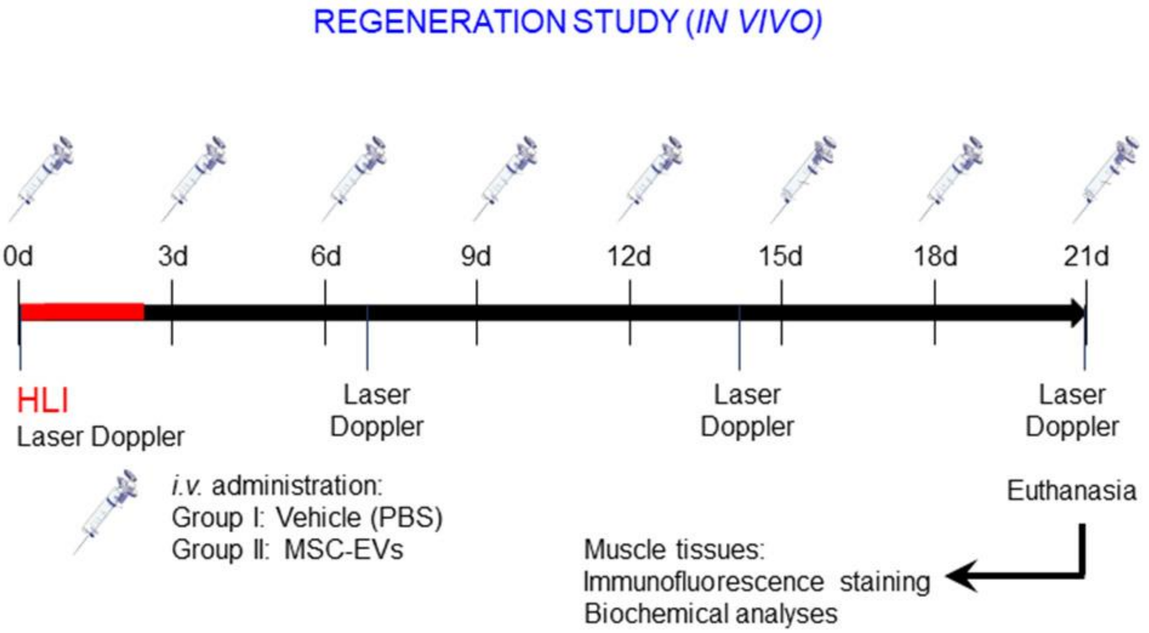

Figure S1. Experimental layout of *in vivo study* (murine model of HLI). Mice underwent critical hind limb ischemia (HLI) by permanent ligation of the left femoral artery (FA) at day 0 (0d). Mice were randomly divided into two (2) groups that were *i.v.* administrated every 3 days for 21 days with: 1) vehicle (PBS, Control group) or 2) MSC-EVs (10 μg/ml of blood). Blood flow in ischemic and non-ischemic limbs was measured before limb injury as well as at 7-, 14- and 21-d post FA occlusion by Laser Doppler perfusion imaging technique. At 21d post HLI, mice underwent euthanasia and muscle tissues were isolated for further biochemical and histological analysis.

Figure S2

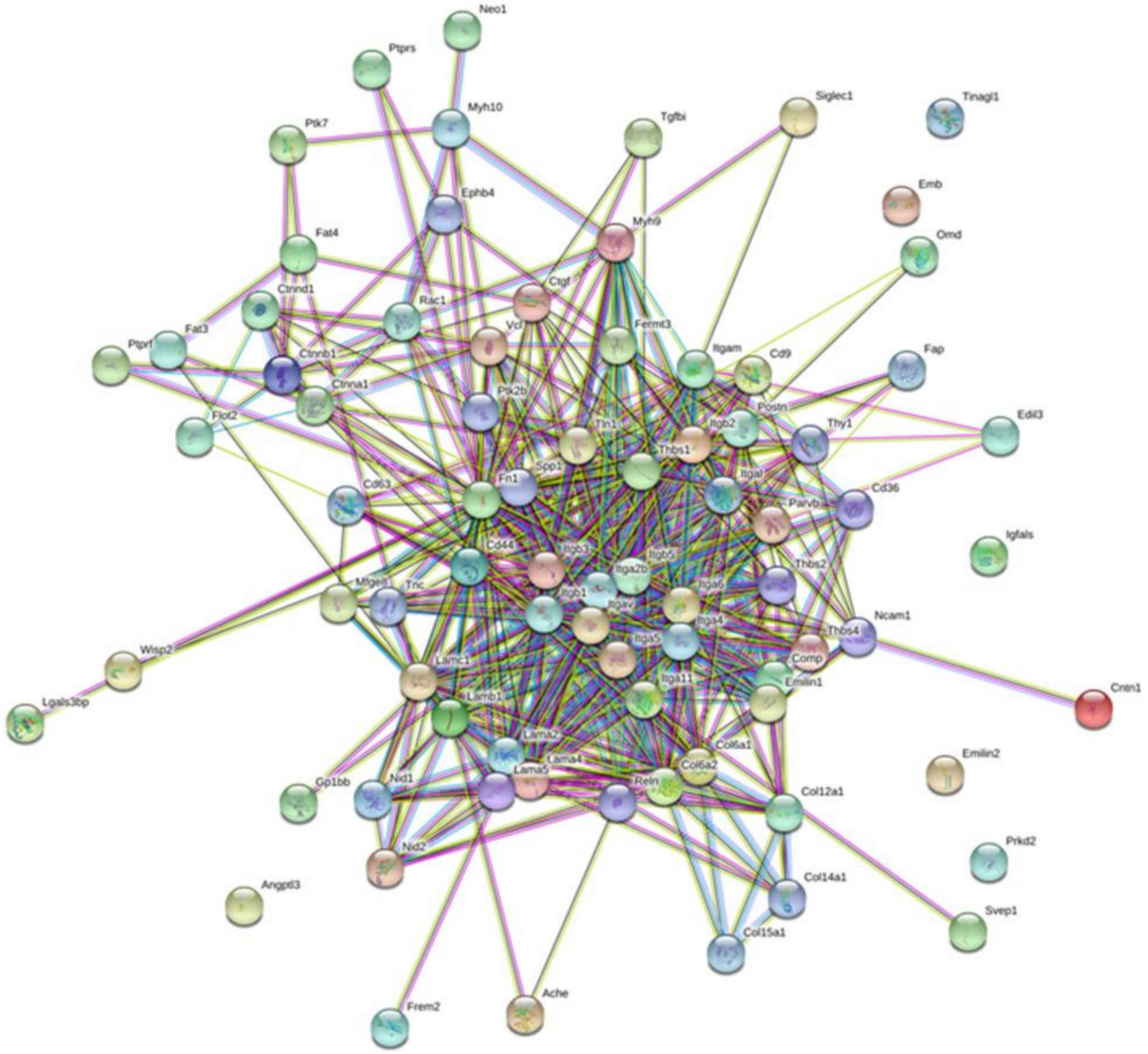

Figure S2. Network of interactions between proteins engaged in cell adhesion found in MSC-EVs by String database (ver 11.0). The number of solid lines between proteins correspond to the strength of interaction. The full name of displayed proteins can be found in UniProt database. List of cell adhesion molecules were included in *Table S3*.

Figure S3

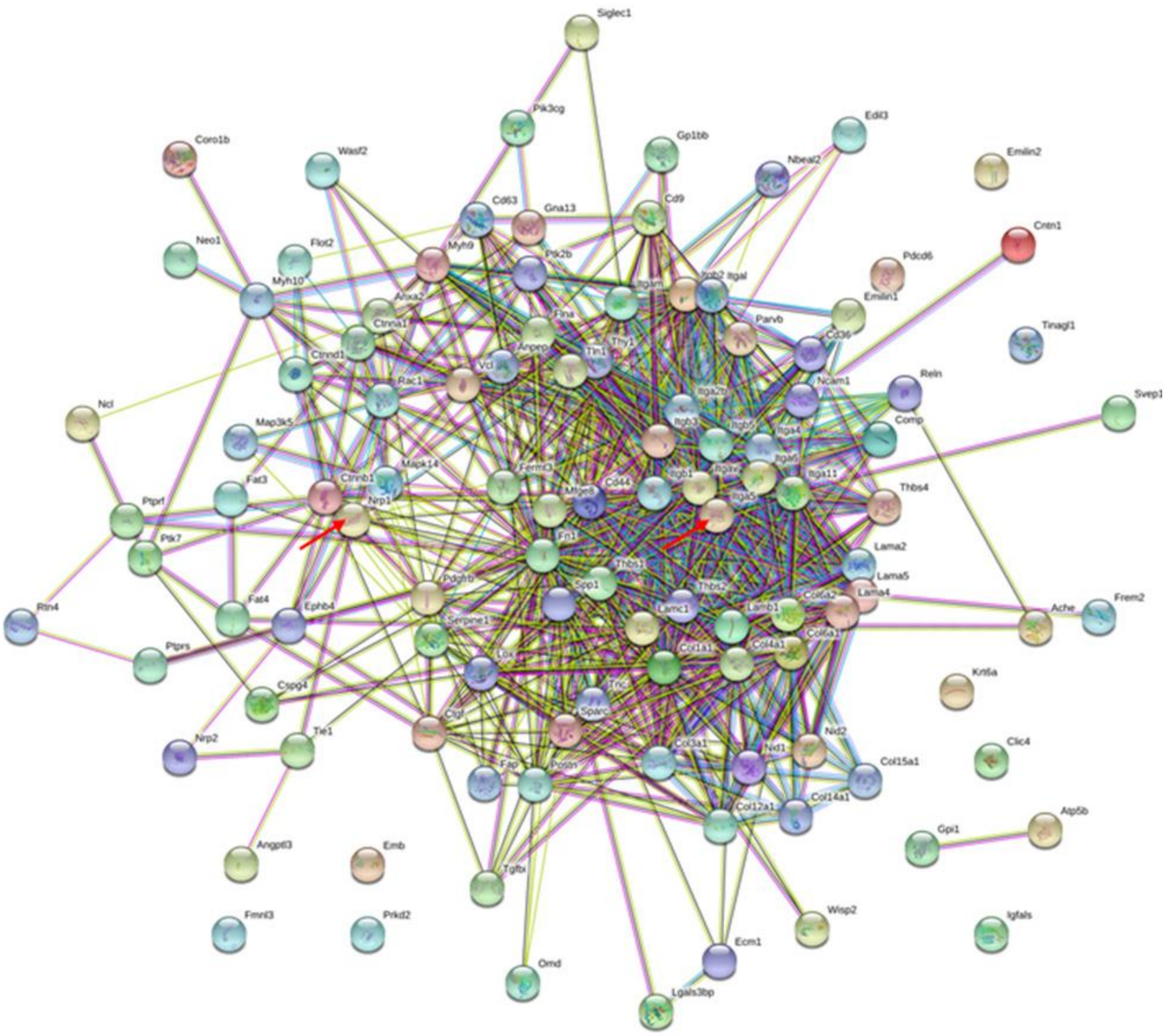

Figure S3. Network of interactions between proteins engaged in <u>cell adhesion, angiogenesis and wound healing</u> found in MSC-EVs by String database (ver 11.0). The number of solid lines between proteins correspond to the strength of interaction. The full name of displayed proteins can be found in UniProt database.

Figure S4

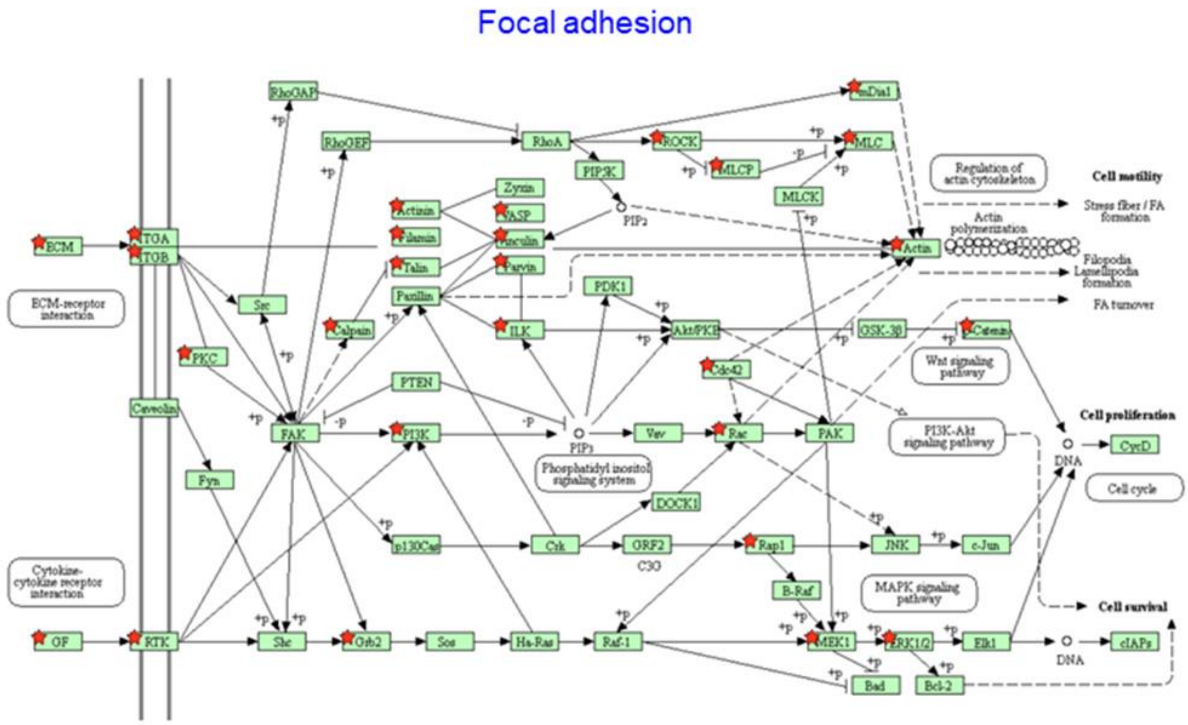

Figure S4. Signaling pathways associated with proteins found in MSC-EVs and related to <u>focal adhesion</u> by DAVID functional annotation bioinformatics microarray analysis. Impact on following biological processes is shown: cell motility, proliferation and survival. Red asterisks indicate proteins identified to be significantly changed in MSC-EVs when compared to MSCs by LC-MS/MS.

Figure S5

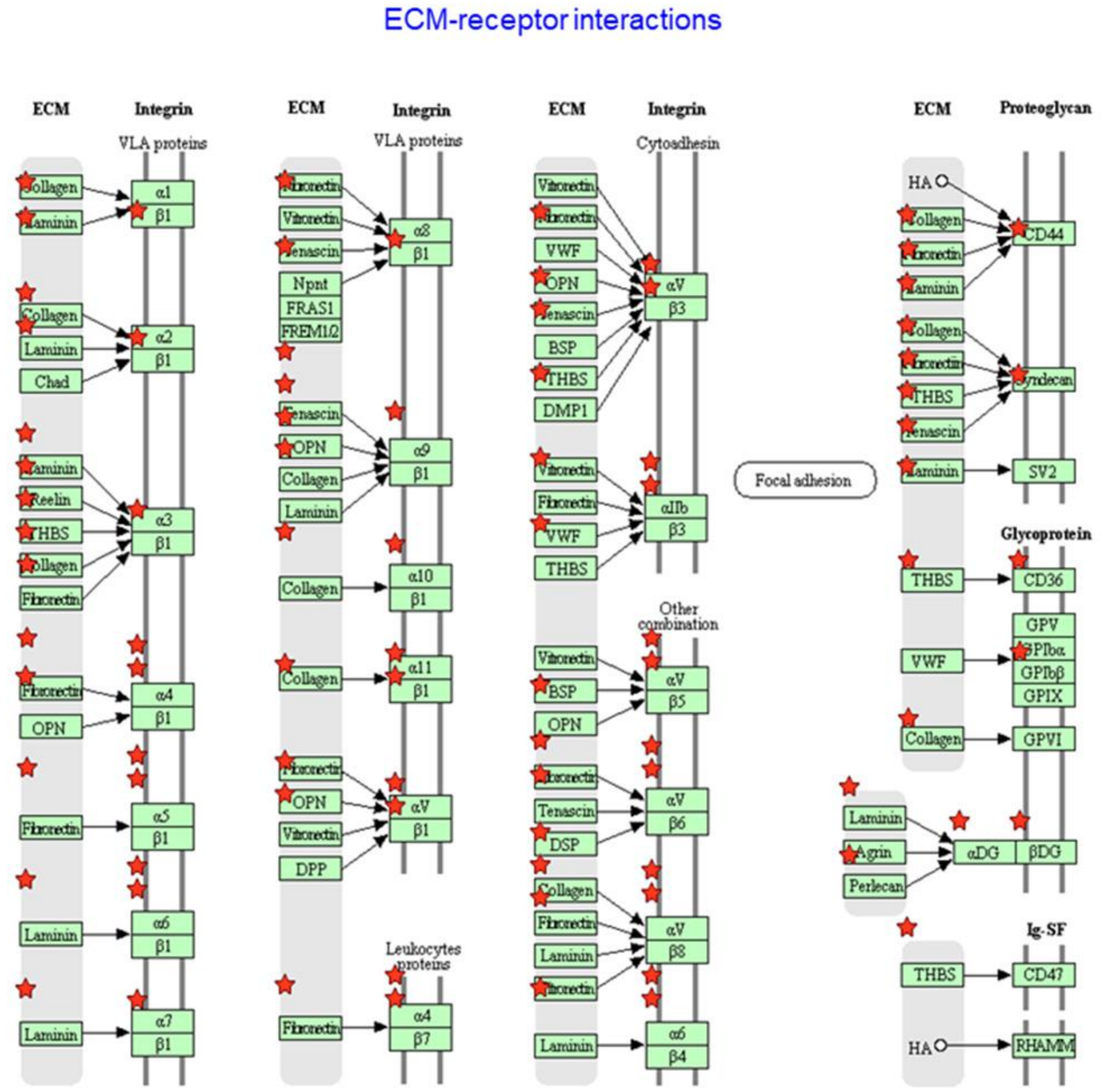

Figure S5. Signaling pathways associated with proteins found in MSC-EVs and related to <u>ECM-receptor interactions</u> by DAVID functional annotation bioinformatics microarray analysis. Red asterisks indicate proteins identified to be significantly changed in MSC-EVs when compared to MSCs by LC-MS/MS.

Figure S6

A

miRNA	$Ct_{MSC\text{-}EVs}$	Ct_{MSCs}	ΔCt ($Ct_{MSC\text{-}EVs} - Ct_{MSCs}$)
**Enriched in MSC-EVs**			
miR-1952	32.1	40.0	-7.9
miR-376a-3p	32.3	40.0	-7.7
miR-182-5p	32.4	40.0	-7.6
miR-668-3p	33.0	40.0	-7.0
miR-1188-5p	35.3	40.0	-4.7
miR-409-5p	35.7	40.0	-4.3
miR-339-3p	36.1	40.0	-3.9
miR-325-3p	36.2	40.0	-3.8
miR-369-3p	36.4	40.0	-3.6
miR-466f-5p	37.7	40.0	-2.3
**Present in MSC-EVs (involved in regulation of pro-regenerative processes)**			
miR-382-5p	27.1	30.7	-3.6
miR-132-3p	29.4	30.8	-1.4
miR-126-3p	31.9	33.2	-1.3

B

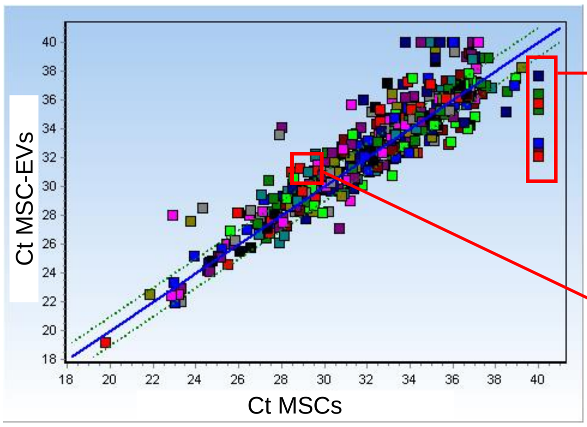

Enriched in MSC-EVs:

miR-1952
miR-376a-3p
miR-182-5p
miR-668-3p
miR-1188-5p
miR-409-5p
miR-339-3p
miR-325-3p
miR-369-3p
miR-466f-5p

Present in MSC-EVs
(involved in regulation of proregenerative processes):

miR-382-5p
miR-132-3p
miR-126-3p

Figure S6. Content of miRNAs in MSC-EVs. (A) Number of amplification cycles (Ct) for miRNAs enriched in MSC-EVs and selected miRNAs present in MSC-EVs. **(B)** (Left) Scatter plot of miRNA expression (Ct) in MSCs (X-axis) and MSC-EVs (Y-axis). Each small square represents one microRNA molecule. (Right) List of miRNAs enriched in MSC-EVs when compared to MSCs and list of selected miRNAs involved in pro-regenerative processes.

Figure S7

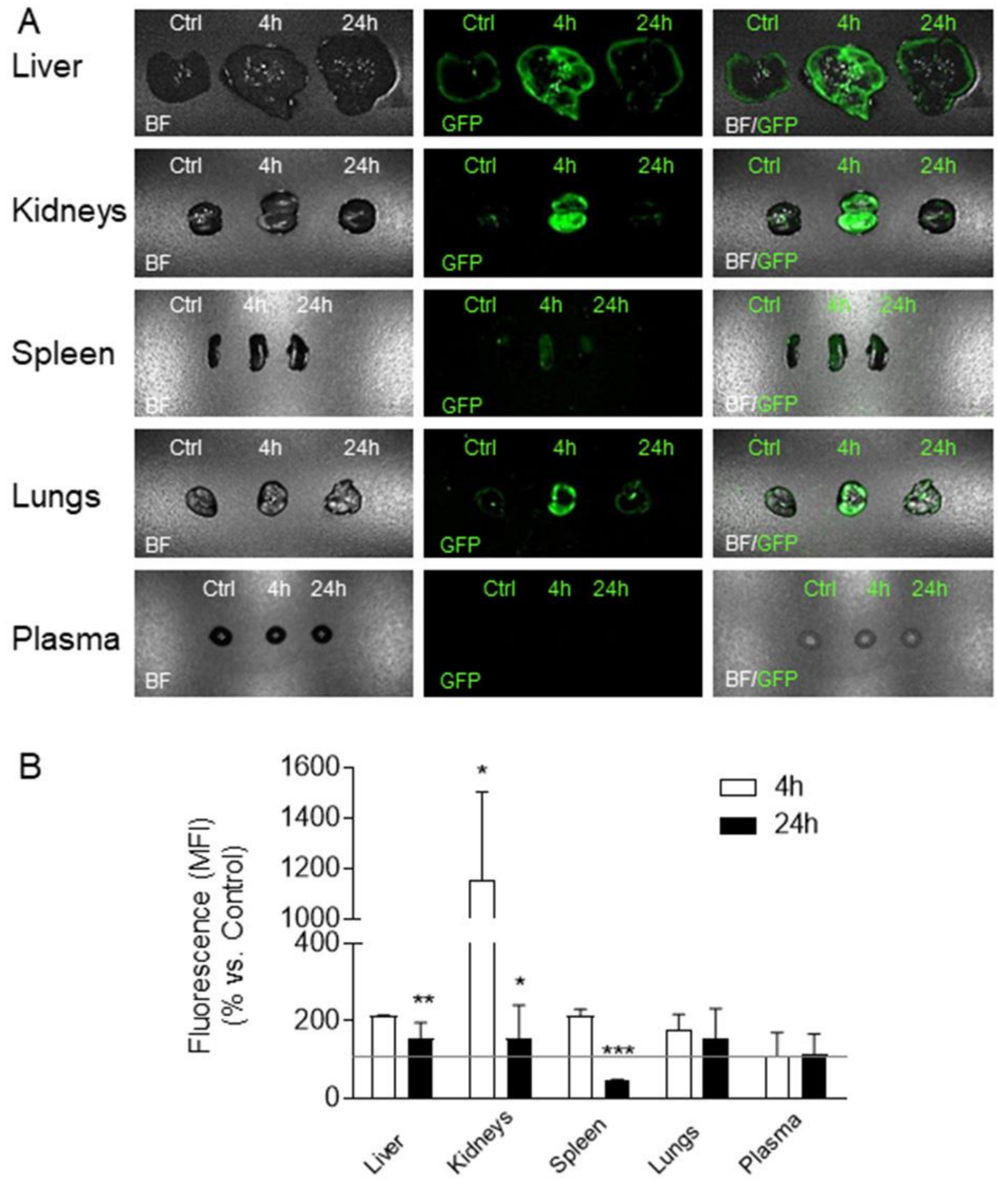

Figure S7. Bio-distribution of systematically delivered MSC-EVs in murine model of hindlimb ischemia (HLI) ***in vivo*****. (A)** Representative images of selected organs (liver, kidneys, spleen, lungs) and plasma at 4 or 24h post *i.v.* injection of: GFP$^+$ MSC-EVs (green) or vehicle (PBS, Control) by MAESTRO In-Vivo fluorescence imaging system. (**B**) Average fluorescence emitted from analyzed organs and plasma at experimental time points. The values of the mean fluorescence intensity (MFI) are presented with reference to values obtained for vehicle- treated mice as Control. MFI is computed as 100% for Control and is indicated by solid line on the graph. Student *t*-test, comparison to vehicle- treated samples (*P<0.05, **P<0.01, ***P<0.001).

Figure S8

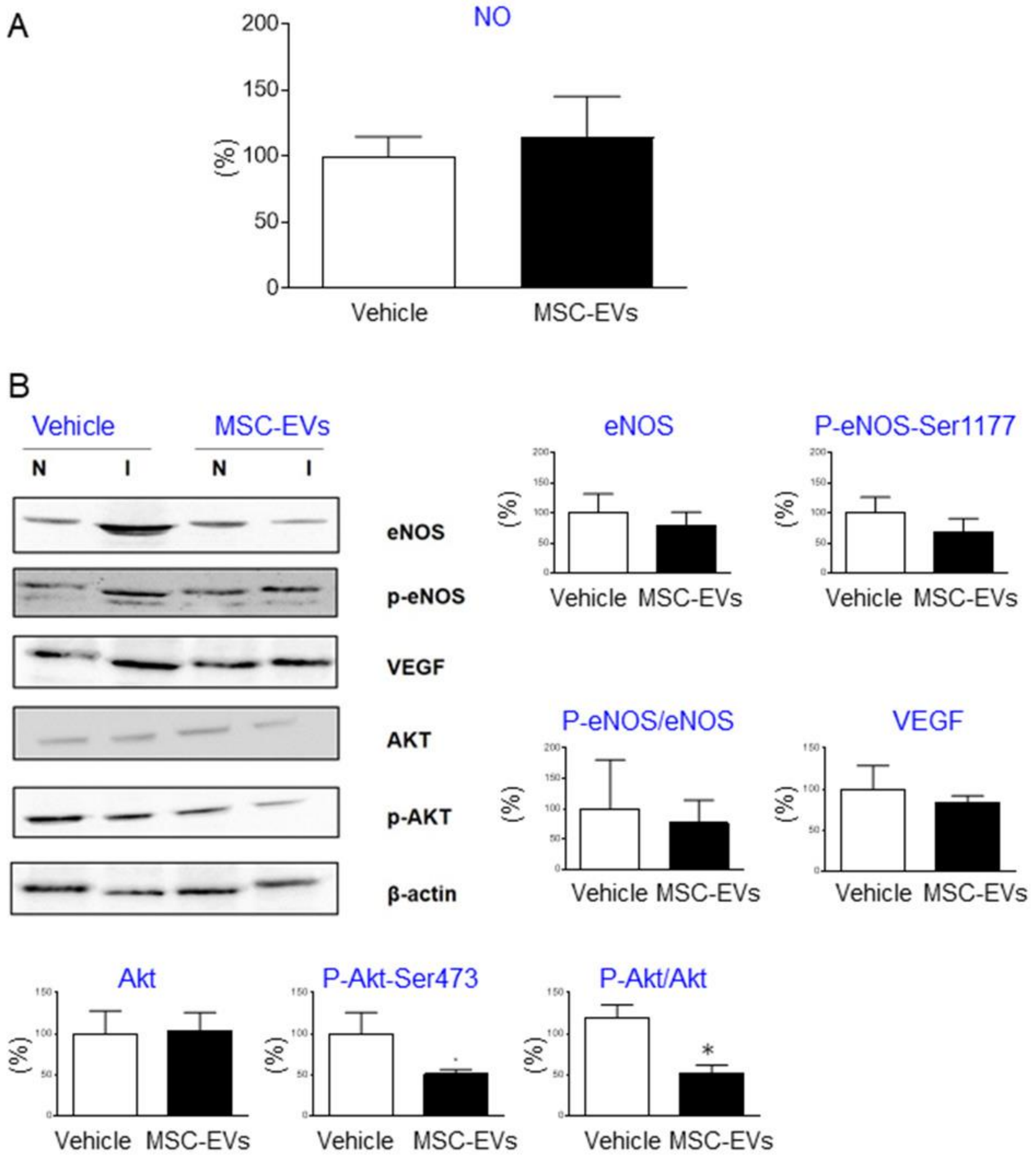

Figure S8. Biochemical analysis of aortas isolated from mice at 21d post HLI. (**A**) Quantification of NO production in aortas by EPR. (**B**) Western immunoblot analysis of activation of proteins regulating pro-regenerative processes including of eNOS, VEGF, AKT and their phosphorylated variants in aortas from vehicle- and MSC-EV- treated animals. (Top Left) Qualitative data by representative Western blot. (Top Right, Bottom) Quantitative data. Values are expressed as a ratio of ischemic (I) to non-ischemic (N) signal amplitude/ protein expression and calculated as percent (%) of signal in control tissues form vehicle (PBS)- treated animals. Mann-Whitney test, comparison to vehicle- treated samples (*P<0.05).

Additional References

1. Théry C, Witwer KW, Aikawa E, Alcaraz MJ, Anderson JD, Andriantsitohaina R, et al. Minimal information for studies of extracellular vesicles 2018 (MISEV2018): a position statement of the International Society for Extracellular Vesicles and update of the MISEV2014 guidelines. J Extracell Vesicles. 2018;7:1535750.

2. Wisniewski JR, Zougman A, Nagaraj N, Mann M, Wi JR. Universal sample preparation method for proteome analysis. Nat Methods. 2009;6:359–62.

3. Wiśniewski JR, Duś K, Mann M. Proteomic workflow for analysis of archival formalin-fixed and paraffin-embedded clinical samples to a depth of 10000 proteins. Proteomics - Clin Appl. 2013;7:225–33.

4. Käll L, Canterbury JD, Weston J, Noble WS, MacCoss MJ. Semi-supervised learning for peptide identification from shotgun proteomics datasets. Nat Methods. 2007;4:923–5.

5. Keerthikumar S, Chisanga D, Ariyaratne D, Al Saffar H, Anand S, Zhao K, et al. ExoCarta: A Web-Based Compendium of Exosomal Cargo. J Mol Biol. 2016;428:688–92.

6. Vlachos IS, Zagganas K, Paraskevopoulou MD, Georgakilas G, Karagkouni D, Vergoulis T, et al. DIANA-miRPath v3.0: Deciphering microRNA function with experimental support. Nucleic Acids Res. 2015;W460–6.

7. Niiyama H, Huang NF, Rollins MD, Cooke JP. Murine Model of Hindlimb Ischemia. J Vis Exp. 2009;1–4.

8. Hoff J. Methods of Blood Collection in the Mouse. Lab Anim (NY). 2000;29:47–53.

9. Couffinhal T, Silver M, Zheng LP, Kearney M, Witzenbichler B, Isner JM. Mouse model of angiogenesis. Am J Pathol. 1998;152:1667–79.

10. Limbourg A, Korff T, Napp LC, Schaper W, Drexler H, Limbourg FP. Evaluation of postnatal arteriogenesis and angiogenesis in a mouse model of hind-limb ischemia. Nat Protoc. 2009;4:1737–48.

11. van Balkom BWM, Gremmels H, Giebel B, Lim SK. Proteomic Signature of Mesenchymal Stromal Cell-Derived Small Extracellular Vesicles. Proteomics. 2019;19:e1800163.
